# Prospective comparison of temporal changes in myocardial function in women with Takotsubo versus anterior STEMI

**DOI:** 10.1007/s00392-025-02633-4

**Published:** 2025-03-20

**Authors:** Sandeep Jha, Angela Poller, Aaron Shekka Espinosa, Linnea Molander, Valentyna Sevastianova, Rickard Zeijlon, Koen Simons, Emanuele Bobbio, Carlo Pirazzi, Andreas Martinsson, Tomas Mellberg, Thorsteinn Gudmundsson, Petronella Torild, Joakim Sundstrom, Erik Axel Andersson, Sigurdur Thorleifsson, Sabin Salahuddin, Ahmed Elmahdy, Tetiana Pylova, Araz Rawshani, Oskar Angeras, Truls Ramunddal, Kristofer Skoglund, Elmir Omerovic, Bjorn Redfors

**Affiliations:** 1 Department of Molecular and Clinical Medicine, Institute of Medicine, University of Gothenburg, Gothenburg, Sweden; 2 Department of Cardiology, Sahlgrenska University Hospital/S, Bruna Straket 16, 431 45 Gothenburg, Sweden; 3 Department of Clinical Physiology, Sahlgrenska University Hospital/S, Gothenburg, Sweden; 4 Department of Internal Medicine, Sahlgrenska University Hospital/S, Gothenburg, Sweden; 5 School of Public Health and Community Medicine, Institute of Medicine, Gothenburg University, Gothenburg, Sweden; 6 Wallenberg Centre for Molecular and Translational Medicine, Institute of Medicine, University of Gothenburg, Gothenburg, Sweden; 7 Clinical Trial Centre, Cardiovascular Research Foundation, New York, USA

**Keywords:** ST-elevation myocardial infarction, Takotsubo syndrome, Echocardiography

## Abstract

**Background:**

Takotsubo syndrome (TS) and STEMI with timely reperfusion are both characterized by reversible acute myocardial dysfunction, often referred to as myocardial stunning. The natural course of cardiac functional recovery is incompletely understood in TS and STEMI. The aim of this study was to prospectively compare changes in cardiac function over the acute and subacute phases in women with TS versus anterior STEMI.

**Methods:**

The Stunning in Takotsubo versus Acute Myocardial Infarction (STAMI) study prospectively enrolled 61 women with TS and 41 women with STEMI. Echocardiography and blood sampling was performed within 4 h of admission and at 1, 2, 3, 7, 14, and 30 days after admission. The primary outcome was the proportion of reversible left ventricular akinesia (defined as extent of akinesia at baseline versus at 30 days) that resolved by 72 h. Secondary outcomes included LVEF, GLS, and TAPSE. Mixed effects linear regression or mixed effects tobit models with random intercepts were used to model echocardiographic parameters over time.

**Results:**

At 72 h 40.4% [95% CI 30.1%, 50.1%] of the reversible akinesia had resolved in women with TS, versus 54.7% [95% CI 38.3%, 72.0%] for STEMI (difference 14.3% [95% CI − 4.6%, 34.3%]). Time-course of recovery of LVEF and GLS was also similar in TS and STEMI. TAPSE was reduced in TS but normal in STEMI; and recovered in a similar timeframe as the left ventricular indices. In both TS and STEMI, considerable recovery of cardiac function occurred after 7 days.

**Conclusions:**

The time course of recovery of cardiac function is similar in TS and STEMI.

**Trial registration:**

ClinicalTrials.gov ID NCT04448639, https://clinicaltrials.gov/study/NCT04448639.

**Graphical abstract:**

The stunning in Takotsubo versus acute myocardial infarction (STAMI) study compared the changes in cardiac function over time in 61 women with takotsubo syndrome (TS) versus 41 women with ST elevation myocardial infarction (STEMI) over a 30-day period. The time course of cardiac recovery was similar in takotsubo and anterior ST-elevation myocardial infarction. In both takotsubo and ST-elevation myocardial infarction, recovery of cardiac function takes on average longer than 7 days.

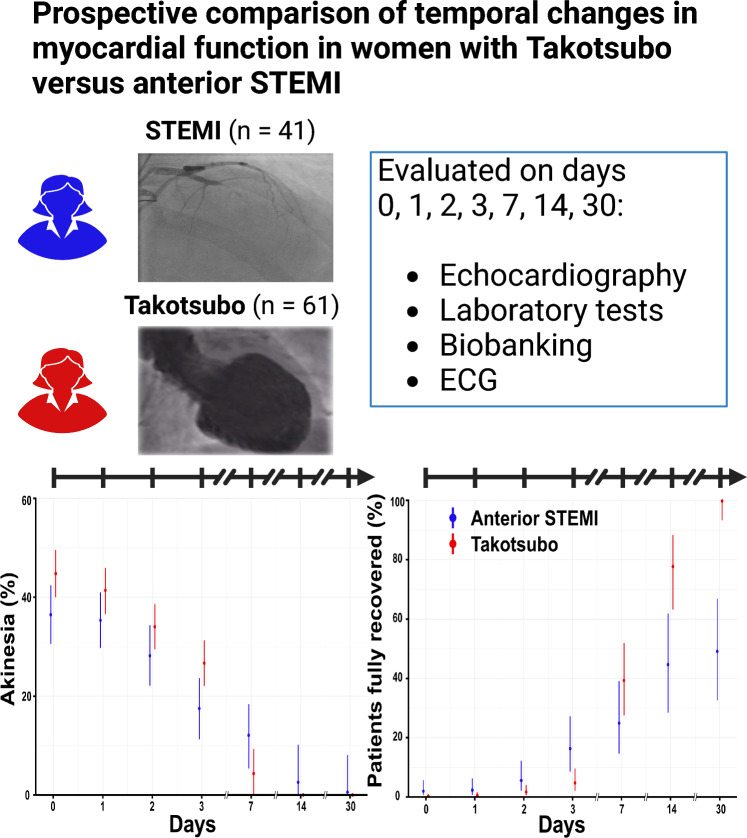

**Supplementary Information:**

The online version contains supplementary material available at 10.1007/s00392-025-02633-4.

## Introduction

Takotsubo syndrome (TS) and ST-elevation myocardial infarction (STEMI) with timely reperfusion are both characterized by reversible acute myocardial dysfunction, often referred to as myocardial stunning [1–3]. However, the natural course of cardiac functional recovery is incompletely understood both in TS and STEMI. We sought to compare cardiac function over time among patients with TS and patients with anterior STEMI undergoing timely reperfusion.

Because the incidence of TS is notably higher among women than men [4–6], and since sex differences in cardiac function after STEMI have been described [7, 8], we limited our study cohort to women. We hypothesized that because TS and STEMI have distinct aetiologies, the recovery of cardiac function over the acute and subacute phase would follow different trajectories in the two conditions.

## Methods

### Study design

The Stunning in Takotsubo versus Acute Myocardial Infarction study (STAMI, NCT04448639) is a prospective, observational study in which patients with TS or STEMI without preexisting cardiac dysfunction undergo echocardiography, electrocardiography, and laboratory assessment within 4 h of admission and at days 1, 2, 3, 7, 14, and 30 (Supplementary tables S1 and S2) during 2019–2024 at Sahlgrenska University Hospital, Gothenburg, Sweden. TS was defined according to the European Society of Cardiology (ESC) diagnostic criteria [9]. Patients with STEMI were required to undergo primary PCI within 6 h of symptom onset. Only women with TS or anterior STEMI were included in the analysis population. All study participants gave informed consent, and the study was approved by the Swedish Ethical Review Authority (Dnr. 2022-01003-02) and carried out in accordance with the Declaration of Helsinki. All data was handled after the EU Data Protection Directive.

### Echocardiography

All echocardiograms were performed according to a detailed echocardiographic protocol by experienced echocardiographers (Supplemental Appendix 1); each echocardiogram was analysed offline per the protocol in a blinded manner independently by two experienced echocardiographers (SJ and AP). In preparation for STAMI, we demonstrated that the intra- and inter-observer variability for the echocardiographic variables of interest is low, and the method is reliable [10]. For each of the echocardiographic variables, the mean value between the two observers were used for analyses.

### Definitions

**Fig. 1:**
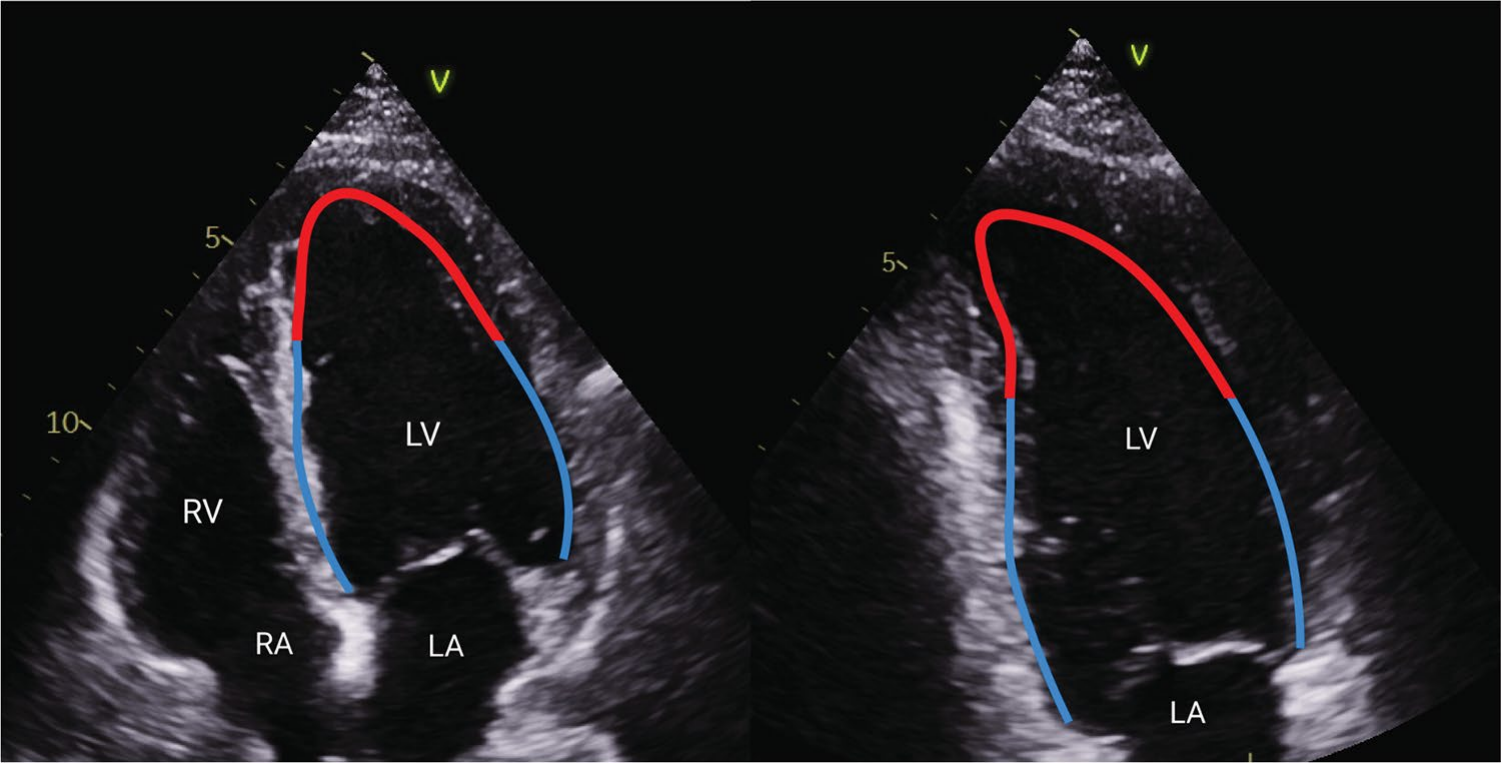
Assessment of the proportion akinesia in the left ventricular four- (left) and two (right) chamber view. First, the left ventricular endocardial border was outlined in apical four chamber view (blue line) in end-diastole. Secondary, the endocardial border and the extent of akinesia was outlined (red line). The total extent of akinesia in millimetres in both the apical four- and two chamber views was divided by the total length in millimetres in both apical four- and two chamber views in diastole multiplied by 100. *RA* right atrium, *RV* right ventricle, *LV* left ventricle, *LA* left atrium

We defined proportion akinesia (PrA) as the proportion of the left ventricular endocardial length (as measured in the apical 2- and 4-chamber views at end-diastole) that was akinetic (Fig. 1) [10]. We defined early akinesia resolution as the difference in PrA between baseline (day 0) and day 3; and total akinesia resolution as the difference in PrA between baseline and day 30.

### Primary outcome

In this study, we defined stunning as reversible akinesia. The primary outcome was stunning resolution at 3 days, defined as early resolution of akinesia divided by total resolution of akinesia per the following formula:

$$stunning \, resolution=\frac{{PrA}_{baseline}-{PrA}_{3days}}{{PrA}_{baseline}-{PrA}_{30days}}$$

The reason for choosing resolution of akinesia rather than LVEF or LV GLS as the primary outcome is that we were primarily interested in studying the recovery of the affected myocardial segments, and remote, unaffected regions of LV can compensate for the dysfunctional myocardial segments and mitigate the reduction in both LVEF and GLS [11].

### Secondary outcomes

Prespecified secondary outcomes of interest were the differences between women with TS and women with STEMI over time in regards to (i) left ventricular ejection fraction (LVEF), (ii) left ventricular global longitudinal strain (GLS), (iii) wall motion score index (WMSI), (iv) tricuspid annular plane systolic excursion (TAPSE), (v) serum troponin-I, (vi) serum troponin-T, (vii) serum troponin-I/troponin-T ratio, (viii) serum NT-proBNP, (ix) serum NT-proBNP/troponin-I ratio, and (x) serum NT-proBNP/troponin-T ratio.

### Statistical analyses

Continuous data are reported as mean ± standard deviation for variables with normal distribution, and median (quartile 1 [Q1], quartile 3 [Q3]) for variables with skewed distribution. Categorical data are reported as frequency (percentage). Missing data in akinesia was partially imputed as follows: observations of 0% akinesia were carried forward if there was no subsequent observation of non-zero akinesia. Patients who experienced a second event during follow up were censored from the day and time of the second event. Second events included additional myocardial infarction and additional revascularisation, including staged PCI.

The primary analysis compared stunning resolution between women with TS and women with anterior STEMI. Identical tobit mixed models were fitted to model the evolution of akinesia over time. The tobit model functions as a zero-inflated and left-censored normal distribution, allowing for zero akinesia. Time was included as a fixed categorical variable, and patient-specific trajectories were modelled using “random” intercepts. TS and STEMI were fitted separately, allowing both condition-specific reference trajectories and differences in the between-patient and within-patient variation. For each condition, the stunning resolution for the median patient at day 3 was derived from the tobit mixed model using 10,000 posterior samples obtained using Markov Chain Monte Carlo (R package brms 2.21.0). The difference in stunning resolution for the median patient was obtained as the simple difference between the two posterior samples. Percentiles were used to calculate 95% Credible Intervals. Covariates were included in the model using cubic splines for age and body mass index (BMI), and as binary variables for diabetes, hypertension, and chronic obstructive pulmonary disease. The adjusted difference in stunning resolution was calculated for the median patient with median BMI, median age, and without comorbidities.

Mixed-effects linear regression was used to model the temporal variation in echocardiographic and laboratory parameters, with time as a fixed categorical effect and random intercepts assigned to each patient ID. The model was used uniformly for variables with a normal distribution (LVEF, GLS, TAPSE) and for those conforming to normality after logarithmic transformation (troponin-I, troponin-T, NT-proBNP, ratios). BMI, age, diabetes, hypertension, and chronic obstructive pulmonary disease were included as covariates in the models. All statistical analyses were performed with R version 4.4.1. Figures were designed using Affinity Designer 2 and Biorender.com.

## Results

### Study population

The STAMI study cohort consisted of 41 women with anterior STEMI and 61 women with TS. Baseline characteristics were similar between groups, but women with STEMI were on average older (Table 1). Chest pain was the presenting symptom in 34/61 (55.7%) women with TS and 40/41 (97.6%) women with STEMI. Among those who reported having chest pain, the pain was most often described as being of ‘pressure’ character (70.6% and 85.0%), constant rather than intermittent 67.6% and 75.0%), and radiating rather than being localized to one point (70.6% and 80.0%), both among women with TS and women with STEMI. Among women with TS, 18/61 (29.5%) reported a somatic trigger, 28/61 (45.9%) reported an emotional trigger and 15/61 (24.6%) could not identify any trigger. Most women in both groups presented in sinus rhythm. Baseline and discharge drugs are presented in Table 2, and procedural drugs are presented in Supplemental Table S3. ECG findings at admission are presented in Supplemental Table S4. Moderate or severe mitral regurgitation occurred more often in patients with TS than in patients with STEMI (9/61 [14.8%] versus 1/41 [2.4%] and 1/61 [1.6%] versus 0/41 [0.0%]). The median (Q1, Q3) duration of the index hospitalization was 5 (4, 8) days for women with TS and 3 (3, 5) days for women with STEMI.

**Table 1:** Key baseline characteristics per group

Variable	Anterior STEMI N = 41	Takotsubo N = 61
Age (years)	70.0 ± 11.1	69.0 ± 10.5
BMI (kg/m^2^)	25.6 [23.9–29.8]	24.8 [22.8–28.9]
Diabetes	4/41 (9.8%)	5/61 (8.2%)
Hypertension	18/41 (43.9%)	28/61 (45.9%)
Hyperlipidemia	5/41 (12.2%)	10/61 (16.4%)
Atrial fibrillation		
Chronic	0/41 (0.0%)	1/61 (1.6%)
Paroxysmal	1/41 (2.4%)	6/61 (9.8%)
No	40/41 (97.6%)	54/61 (88.5%)
Chronic obstructive pulmonary disease	4/41 (9.8%)	6/61 (9.8%)
Peripheral vascular disease	1/41 (2.4%)	4/61 (6.6%)
Chronic renal disease	0/41 (0.0%)	2/60 (3.3%)
Prior stroke	1/41 (2.4%)	4/61 (6.6%)
Prior stroke type		
Ischemic	0/1 (0.0%)	4/4 (100.0%)
Unknown	1/1 (100.0%)	0/4 (0.0%)
Smoking status		
Current smoker (within 30 days)	15/41 (36.6%)	8/61 (13.1%)
Former smoker (more than 30 days ago)	9/41 (22.0%)	18/61 (29.5%)
Takotsubo trigger		
Somatic	NA	18/61 (29.5%)
Emotional	NA	28/61 (45.9%)
None	NA	15/61 (24.6%)
Chest pain	40/41 (97.6%)	34/61 (55.7%)
Time from first pain (min)	283 [156–1203]	334 [100–1215]
Time from worst pain (min)	136 [89–180]	100 [79–135]
Time from contact pain (min)	147 [105–186]	105 [100–334]
Pain character		
Aching	4/40 (10.0%)	8/34 (23.5%)
Burning	0/40 (0.0%)	2/34 (5.9%)
Pressure	34/40 (85.0%)	24/34 (70.6%)
Stabbing	2/40 (5.0%)	0/34 (0.0%)
Pain persistence		
Constant pain	30/40 (75.0%)	23/34 (67.6%)
Intermittent—mostly pain	7/40 (17.5%)	8/34 (23.5%)
Intermittent—mostly pain free	3/40 (7.5%)	3/34 (8.8%)
Pain localization		
Localized to one point	8/40 (20.0%)	10/34 (29.4%)
Radiating	32/40 (80.0%)	24/34 (70.6%)
Pain intensity (Numerical Rating Scale [33])		
1	0/39 (0.0%)	0/32 (0.0%)
2	0/39 (0.0%)	0/32 (0.0%)
3	0/39 (0.0%)	3/32 (9.4%)
4	1/39 (2.6%)	2/32 (6.3%)
5	4/39 (10.3%)	5/32 (15.6%)
6	1/39 (2.6%)	4/32 (12.5%)
7	6/39 (15.4%)	5/32 (15.6%)
8	13/39 (33.3%)	3/32 (9.4%)
9	3/39 (7.7%)	5/32 (15.6%)
10	11/39 (28.2%)	5/32 (15.6%)
Pain localization		
Localized to one point	8/40 (20.0%)	10/34 (29.4%)
Radiating	32/40 (80.0%)	24/34 (70.6%)
Radiating		
Left arm	22/32 (68.8%)	15/24 (62.5%)
Right arm	18/32 (56.3%)	6/24 (25.0%)
Neck and jaw	11/32 (34.4%)	12/24 (50.0%)
Back	9/32 (28.1%)	13/24 (54.2%)
Abdomen	2/32 (6.3%)	4/24 (16.7%)
Other symptoms		
Nausea	22/41 (53.7%)	23/61 (37.7%)
Vertigo	13/41 (31.7%)	20/61 (32.8%)
Cold sweat	25/41 (61.0%)	21/61 (34.4%)
Dyspnoea	14/41 (34.1%)	33/61 (54.1%)
Anxiety	7/41 (17.1%)	26/61 (42.6%)
Fatigue	6/41 (14.6%)	18/61 (29.5%)
Heart rhythm admission		
Sinus	40/41 (97.6%)	55/58 (94.8%)
Atrial fibrillation or flutter	1/41 (2.4%)	1/58 (1.7%)
Pacemaker	0/41 (0.0%)	2/58 (3.4%)
Heart rate at admission (BPM)	76 (71, 90)	99 (76, 111)
SBP at admission (mmHg)	129 (114, 145)	129 (115, 149)
DBP at admission (mmHg)	81 (69, 90)	84 (71, 94)
Killip class admission		
I	32/41 (78.0%)	41/60 (68.3%)
II	5/41 (12.2%)	11/60 (18.3%)
III	3/41 (7.3%)	5/60 (8.3%)
IV	1/41 (2.4%)	3/60 (5.0%)
Oxygen at admission	9/41 (22.0%)	18/60 (30.0%)
Culprit vessel		
Left main	1/41 (2.4%)	NA
Proximal LAD	24/41 (58.5%)	NA
Mid LAD	13/41 (31.7%)	NA
Distal LAD	1/41 (2.4%)	NA
D1	2/41 (4.9%)	NA
Takotsubo phenotype		
Apical ballooning	NA	56/61 (91.8%)
Midventricular ballooning	NA	2/61 (3.3%)
Biventricular ballooning	NA	3/61 (4.9%)

Data are shown as n (%), mean ± standard deviation, or median [interquartile range]

*BMI* body mass index, *BPM* beats per minute, *DBP* diastolic blood pressure, *LAD* left anterior descending, *SBP* systolic blood pressure

**Table 2:** Drugs at baseline and discharge

Baseline drugs	Anterior STEMI N = 41	Takotsubo N = 61
Antidiabetic		
Metformin	3/4 (75.0%)	4/5 (80.0%)
SGLT2 inhibitor	1/4 (25.0%)	2/5 (40.0%)
Other antidiabetics	1/4 (25.0%)	2/5 (40.0%)
Corticosteroids	3/41 (7.3%)	6/61 (9.8%)
Paracetamol	4/41 (9.8%)	7/61 (11.5%)
ASA	3/41 (7.3%)	10/61 (16.4%)
P2Y12i	1/41 (2.4%)	2/61 (3.3%)
P2Y12i type		
Clopidogrel	1/1 (100.0%)	2/2 (100.0%)
Oral anticoagulants	2/41 (4.9%)	7/61 (11.5%)
Oral anticoagulants type		
Apixaban	2/2 (100.0%)	6/7 (85.7%)
Rivaroxaban	0/2 (0.0%)	1/7 (14.3%)
Long-acting nitrates	2/41 (4.9%)	0/61 (0.0%)
MRA	0/41 (0.0%)	0/61 (0.0%)
Beta blocker	10/41 (24.4%)	17/61 (27.9%)
ACEi	1/41 (2.4%)	5/61 (8.2%)
ARB	8/41 (19.5%)	18/61 (29.5%)
SGLT2 inhibitor	1/41 (2.4%)	2/61 (3.3%)
Calcium channel blocker	5/41 (12.2%)	8/61 (13.1%)
Calcium channel blocker type		
Dihydropyridine	5/5 (100.0%)	8/8 (100.0%)
Loop diuretic	0/41 (0.0%)	2/61 (3.3%)
Thiazide diuretic	2 /41 (4.9%)	9/61 (14.8%)
Statin	6/41 (14.6%)	15/61 (24.6%)
Ezetrol	1/41 (2.4%)	1/61 (1.6%)
Other lipid lowering drugs	0/41 (0.0%)	1/61 (1.6%)

Discharge drugs	Anterior STEMI N = 41	Takotsubo N = 61
ASA taken throughout 30 days	34/41 (82.9%)	24/61 (39.3%)
P2Y12i taken throughout 30 days	40/41 (97.6%)	5/61 (8.2%)
Beta blocker administrated within 30 days	36/41 (87.8%)	35/61 (57.4%)
ACEi or ARB administrated within 30 days	38/41 (92.7%)	32/61 (52.5%)
Neprilysin inhibitor administrated within 30 days	1/41 (2.4%)	0/61 (0.0%)
Statin administrated within 30 days	40/41 (97.6%)	24/61 (39.3%)
Oral anticoagulants administrated within 30 days	8/41 (19.5%)	18/61 (29.5%)
Long-acting nitrates administrated within 30 days	1/41 (2.4%)	0/61 (0.0%)

*ASA* acetylsalicylic acid, *ACEi* angiotensin-converting enzyme inhibitor, *ARB* angiotensin receptor blocker, *MRA* mineralocorticoid receptor antagonist, *P2Y12i* P2Y12 inhibitor. Data is shown as n (%)

### Primary outcome

**Fig. 2:**
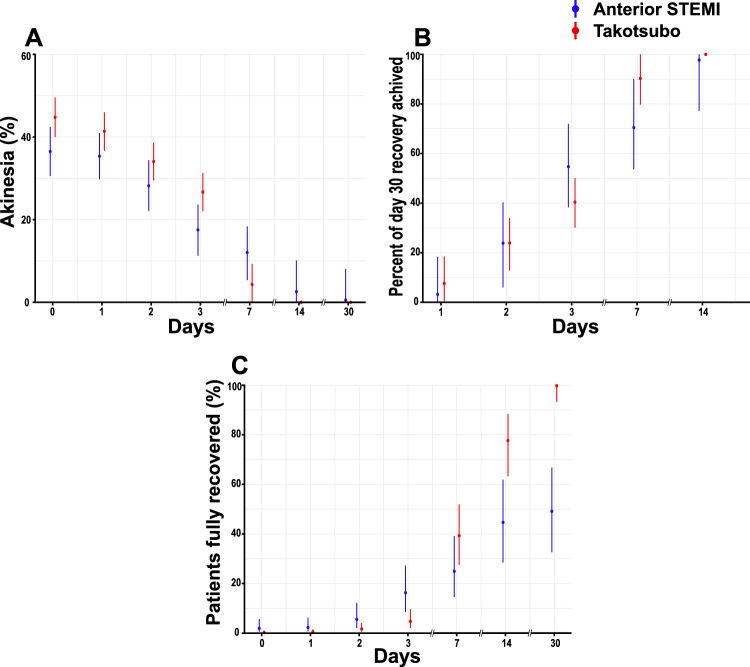
Temporal changes in the extent of left ventricular akinesia. Left ventricular akinesia observed at each day over the course of the study period. The percentage of left ventricular akinesia was calculated in apical 2- and 4-chamber views as 100% times the endocardial length of the akinetic myocardium divided by the total LV endocardial length (**A**). Proportion of the total akinesia recovery (defined as the akinesia at day 0 minus the akinesia at day 30 [i.e. persisting akinesia]) by each day (**B**). Proportion of the akinesia observed on day 0 that resolved (i.e. had 0% akinesia) by each day (**C**). Data shown as the median patients using a tobit mixed model. Lines represent 95% credible intervals

The primary outcome (stunning resolution on day 3) was not statistically significant different between women with TS and women with anterior STEMI in the median patient (TS 40.4% [95% CI 30.1%, 50.1%] vs. STEMI 54.7% [95% CI 38.3%, 72.0%], difference 14.3% [95% CI − 4.6%, 34.3%]) (Fig. 2, Supplemental Table S5). These results were consistent after multivariable adjustment (adjusted difference 17.0% [95% CI − 2.6%, 38.0%]). In a sub-analysis based on age, stunning resolution were similar in patients below the age of 70 (Suppl. Figure 1A–B) and patients ≥ 70 years of age (Suppl. Fig. C–D). All women with TS recovered to 0% akinesia by day 30 whereas 49.1% of women with anterior STEMI recovered to 0% akinesia by day 30 (Fig. 2C, Supplemental Table S5).

### Secondary outcomes

**Fig. 3:**
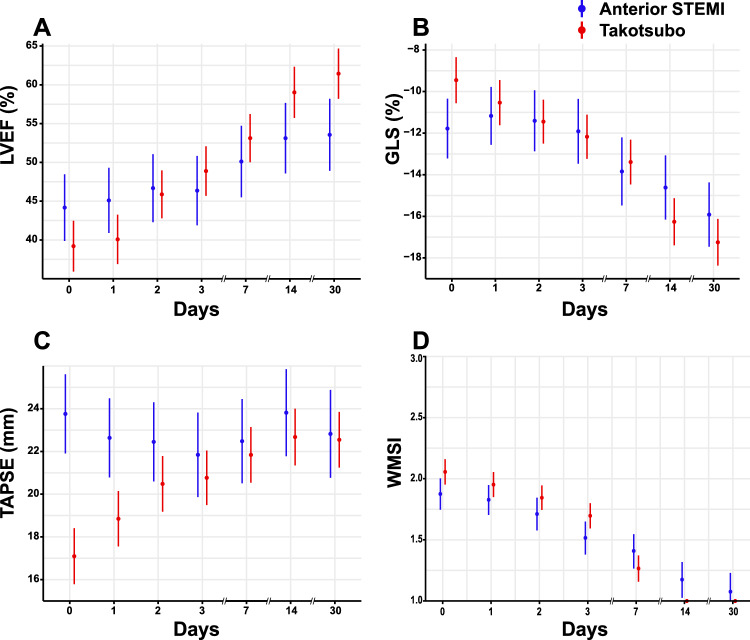
Secondary echocardiographic outcomes. Secondary echocardiographic outcomes include measurements of left ventricular ejection fraction (**A**), global longitudinal strain (**B**), tricuspid annular plane systolic excursion (**C**), and wall motion score index (**D**). Mixed effect models with points representing mean and point range representing 95% confidence intervals (**A-C**) or tobit mixed model showing the median patient with lines representing 95% credible intervals (**D**). *LVEF* left ventricular ejection fraction, *GLS* global longitudinal strain, *TAPSE* tricuspid annular plane systolic excursion, *WMSI* wall motion score index

**Fig. 4:**
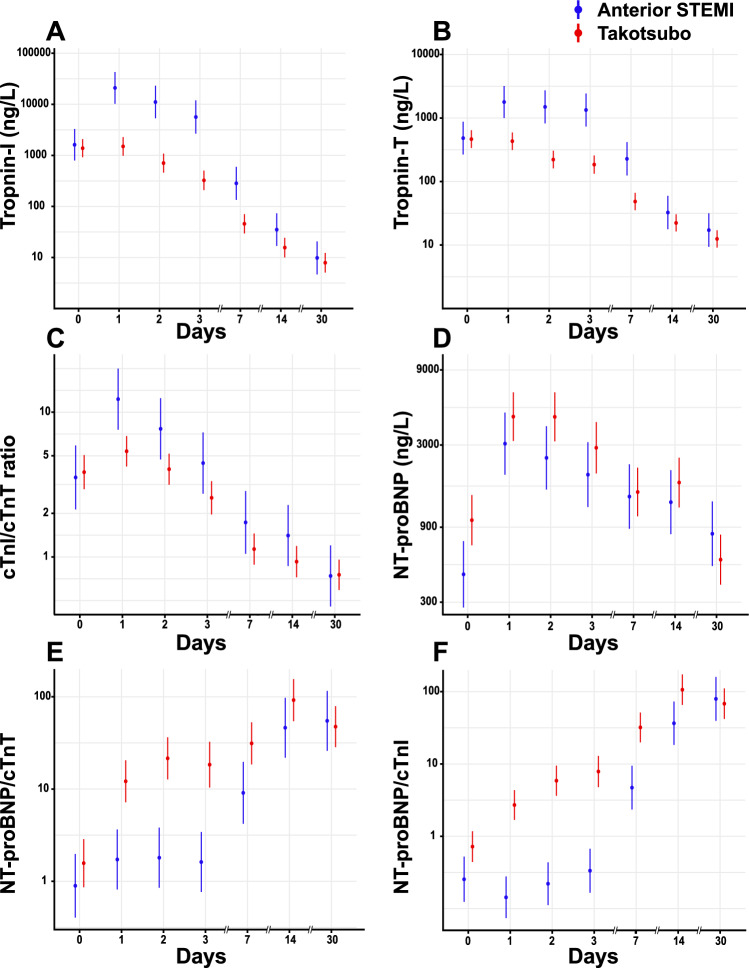
Secondary laboratory outcomes. Secondary laboratory outcomes include serum troponin-I (**A**), serum troponin-T (**B**), the troponin-I to troponin-T ratio (**C**), serum NT-proBNP (**D**), serum NT-proBNP/troponin-T ratio (**E**), and serum NT-proBNP/troponin-I ratio (**F**). Mixed effect models with points representing mean and point-range representing 95% confidence intervals. *cTnI* cardiac troponin-I, *cTnT* cardiac troponin-T

Women with TS had a greater improvement than women with STEMI in echocardiographic parameters of left ventricular function. For both women with TS and women with STEMI, left ventricular function continued to improve more than 7 days after admission (Fig. 3A, B, D, Supplemental Table S5). These results were consistent in both younger (Suppl. Fig. 1A–B) and older patients (Suppl. Fig. 1C–D). Women with TS more often had reduced TAPSE at admission than women with STEMI, but subsequently recovered (Fig. 3C, Supplemental Table S5). Peak troponin-I and peak troponin-T were both lower in women with TS versus women with STEMI (Fig. 4A, B, Supplemental Table S6). The peak ratio between troponin-I and troponin-T was also lower for women with TS versus STEMI (Fig. 4C, Supplemental Table S6). In contrast, NT-proBNP and the ratio of NT-proBNP to troponin-T and troponin-I tended to be higher in women with TS than in women with STEMI (Fig. 4D–F, Supplemental Table S6).

### Clinical outcomes

Short-term clinical outcomes are presented in Table 3. A total of 9 women with TS fulfilled criteria for cardiogenic shock during their index hospitalization (compared to 5 patients with STEMI). No deaths occurred among women with TS or STEMI within 30-days of index event.

**Table 3:** Clinical outcomes

Clinical outcomes	Anterior STEMI N = 41	Takotsubo N = 61
Met criteria for cardiogenic shock^a^ within 3 days	5/41 (12.2%)	9/61 (14.8%)
Death within 30 days	0/41 (0.0%)	0/61 (0.0%)
Reinfarction within 30 days	1/41 (2.4%)	0/61 (0.0%)
Rehospitalization within 30 days	1/41 (2.4%)	0/61 (0.0%)
Stroke or TIA within 30 days	0/41 (0.0%)	0/61 (0.0%)
Thromboembolisation within 30 days	0/41 (0.0%)	0/61 (0.0%)

Data are shown as n (%)

*TIA* transient ischemic attack

^a^Defined as systolic blood pressure below 90 mmHg and signs of heart failure

## Discussion

STAMI is the first study to compare the changes over time in cardiac function during the acute and subacute phases of TS and STEMI. The main findings of the study are that among women without preexisting cardiac dysfunction: (i) left ventricular recovery, while being more pronounced in TS than STEMI, follows a similar time course in TS and STEMI, without obvious differences in the rate of LV recovery between the two conditions; (ii) reversible right ventricular dysfunction is considerably more common among women with TS than women with anterior STEMI, and its recovery tracks that of the LV; and (iii) in both TS and STEMI, considerable additional recovery of cardiac function can be expected after 7 days, suggesting that left ventricular indices obtained at discharge are poor surrogates of intermediate and longer term cardiac function.

We found that the time course of recovery of cardiac function was similar in women with TS and women with STEMI. This observation suggests that at least some of the mechanisms behind cardiac dysfunction may be similar in the two conditions. Whereas reversible cardiac dysfunction in STEMI is the consequence of sublethal acute myocardial ischemia and has been the subject of considerable research efforts, its precise mechanisms are still incompletely understood [12, 13]. Even less is known about the mechanisms behind reversible cardiac dysfunction in TS. The different hypothesized mechanisms for cardiac dysfunction in TS include mechanisms closely related to myocardial ischemia (e.g., microvascular spasm and left ventricular outflow tract obstruction) as well distinctly different mechanisms (such as direct catecholamine effects on the cardiomyocytes) [5, 14–16]. Whether the similarities in the time course of recovery of cardiac function after TS and STEMI observed in STAMI reflect similarities in the underlying mechanisms remains to be determined.

Our study shows that, contrary to women with anterior STEMI, women with TS frequently have depressed right ventricular function. This may be due to the unique pathophysiology of TTS, which can lead to diffuse myocardial stunning, directly affecting not only the left but also the right ventricle [17]. Based on our findings, the presence of concomitant RV dysfunction alongside typical LV dysfunction increases the likelihood of a TTS diagnosis. However, we emphasize that urgent coronary angiography remains essential to exclude coronary occlusion and ongoing myocardial infarction [9]. Despite pronounced impairment of LV functional indices as well as right ventricular engagement, there were no deaths within 30 days among the women with TS in STAMI. This finding is consistent with several prior studies that have reported favourable prognosis for patients with TS despite pronounced cardiac dysfunction [18–20]. In this regard, it is worth noting that all 9 patients with TS that fulfilled the criteria for cardiogenic shock survived. This is also consistent with prior reports that patients with TS often tolerate very low systemic blood pressure without developing progressive multiorgan dysfunction [21–23]. It is also important to note that inclusion in the study required patients to provide informed consent. As a result, patients who developed TS following severe conditions associated with impaired consciousness or those requiring mechanical ventilation were not eligible for enrollment. This inherent selection criterion may have led to the exclusion of more critically ill individuals who have been included in other TS cohorts [24].

Compared to women with anterior STEMI, women with TS more often presented without chest pain, an observation that has been reported before and may be related to the distinct pathomechanisms in STEMI and TS [25]. The higher incidence of anxiety in women with TS is also consistent with prior reports showing an association between anxiety and other psychiatric conditions and TS [26].

Lastly, we found that both for women with TS and for women with STEMI there was still considerable recovery of cardiac function after 7 days. Left ventricular function assessed during the index hospitalization is thus a poor surrogate of intermediate term cardiac function in both conditions. This finding has clinical implications. Both for patients with TS and STEMI, clinical follow-up should be planned at a time when LV function is expected to have recovered, to allow for accurate assessment of long-term cardiac function. This may be particularly important for patients with STEMI, since current treatment guidelines rely on LV function as the basis of several recommendations. Current treatment guidelines from the European Society of Cardiology (ESC) recommend that LVEF is determined before hospital discharge in patients with STEMI; but make no recommendation regarding the timing of the assessment [27]. Our results support the notion that the timing of assessment of LV function must be considered, and that in patients with impaired LV function after STEMI, LV assessment should be repeated at least one week after PCI. In light of our results, it is also worth noting that several clinical trials that have intended to study patients with impaired LV function after STEMI, and which provide the evidence base for treatment recommendations, have enrolled patients based on LV assessment at any time within the first days or weeks after STEMI. Since LV function may differ considerably early after STEMI versus a few days later, the variability in the timing of the LV functional assessment introduces uncertainty regarding exactly what patient population was being studied in these trials [28, 29]. In the present study, the majority of the patients with anterior STEMI were prescribed beta blockers at discharge. Since most of these patients had recovered to a LVEF greater than 50% at 30 days it is uncertain whether they would benefit from long-term treatment with beta blockers, given the lack of benefit of beta blockers for patients with LVEF over 50% in the recent Randomized Evaluation of Decreased Usage of Beta-Blockers after Acute Myocardial Infarction (REDUCE-AMI) trial, long-term treatment with beta blockers is necessary for these patients. The role of beta blockers, as well as the role of other heart failure drugs, is uncertain in patients with TS [30–32]. In this study, beta blockers were prescribed to half of the patients with TS, as was angiotensin converting enzyme inhibitors or angiotensin receptor blockers. Given the transient nature of left ventricular dysfunction in TTS and the absence of robust clinical trial data, further research is needed to clarify whether these medications provide meaningful long-term benefits in this population.

## Limitations

This study has limitations. First, although the study is the largest prospective study of cardiac function in women with TS and STEMI, its sample size is relatively small, and the study is underpowered to detect smaller differences between women with TS versus STEMI in the trajectories of the different cardiac function indices over time. Second, although most patients complied with the study protocol and no patient died or exited the study, not all patients underwent echocardiographic examinations at all timepoints. However, the mixed effects models used in our analysis are capable of handling missing data under the missing-at-random assumption. Third, the lack of cardiac magnetic resonance imaging precludes us from ruling out concomitant infarction or ischemic injury in the patients with TS. Fourth, our study population consisted of a relatively low-risk cohort of women without pre-existing cardiac dysfunction who presented with either a first-time STEMI with timely reperfusion or TS and excluded patients who were unconscious, including those who required sedation due to the need for mechanical ventilation. Lastly, the restriction of the study population to women precludes us from drawing conclusions about changes in cardiac function after TS and STEMI in men.

## Conclusions

The STAMI study shows that among women without preexisting cardiac dysfunction left ventricular recovery follows a similar time course in TS and anterior STEMI, with considerable additional recovery of cardiac function occurring after 7 days in both conditions. Right ventricular function was more often affected in TS than anterior STEMI.

## Supplementary Information

Below is the link to the electronic supplementary material.

Supplementary file1 (DOCX 227 KB)

Supplementary file2 (DOCX 6729 KB)

## Data Availability

The data behind the conclusions of this study are available from the authors upon reasonable request*.* The code for the mixed effects tobit models is available in the following repository: https://bitbucket.org/simonskoen/stami/src/master/.

## Abbreviations

ECG: Electrocardiography
GLS: Global longitudinal strain
LVEF: Left ventricular ejection fraction
MI: Myocardial infarction
PCI: Percutaneous coronary intervention
PrA: Proportion akinesia
STEMI: ST-elevation myocardial infarction
TAPSE: Tricuspid annular plane systolic excursion
TnI: Troponin-I
TnT: Troponin-T
TS: Takotsubo syndrome
WMSI: Wall motion score index
