# Supplementary material for: Prospective comparison of temporal changes in myocardial function in women with Takotsubo versus anterior STEMI
Source: Clin Res Cardiol. 2025 Mar 20;114(12):1705–17. doi: 10.1007/s00392-025-02633-4 (PMC12708765; doi:10.1007/s00392-025-02633-4)
Supplement: Supplementary file 1 — Supplementary file1 (DOCX 227 KB) [file 392_2025_2633_MOESM1_ESM.docx]

**Prospective comparison of temporal changes in myocardial function in women with Takotsubo versus women with anterior STEMI**

**Supplementary material**

Sandeep Jha MD^a,b,f^, Angela Poller MSc^a,c,f^, Aaron Shekka Espinosa MD^a,f^, Linnea Molander MD^a,f^, Valentyna Sevastianova MD PhD^a,f^, Rickard Zeijlon MD PhD^a,d,f^, Koen Simons PhD^a,e,f^, Emanuele Bobbio MD^b^, Carlo Pirazzi MD PhD^b^, Andreas Martinsson MD PhD^b^, Tomas Mellberg MD PhD^b^, Thorsteinn Gudmundsson MD^b^, Petronella Torild RN^b^, Joakim Sundstrom MD^b^, Erik Axel Andersson PhD^a,f^, Sigurdur Thorleifsson MD^a,b,f^, Sabin Salahuddin MD^a,f^, Ahmed Elmahdy MD^a,f^, Tetiana Pylova MD PhD^a,f^, Araz Rawshani MD PhD^a,b,f^, Oskar Angeras MD PhD^a^_,_^b^, Truls Ramunddal MD PhD^a,b^, Kristofer Skoglund MD PhD^b^, Elmir Omerovic MD PhD^a,b^, Bjorn Redfors MD PhD^a,b,f,g^

**AUTHOR AFFILIATIONS:**

^a^Department of Molecular and Clinical Medicine, Institute of Medicine, University of Gothenburg, Gothenburg, Sweden

^b^Department of Cardiology, Sahlgrenska University Hospital/S, Gothenburg, Sweden

^c^Department of Clinical Physiology, Sahlgrenska University Hospital/S, Gothenburg, Sweden

^d^Department of Internal Medicine, Sahlgrenska University Hospital/S, Gothenburg, Sweden

^e^School of Public Health and Community Medicine, Institute of Medicine, Gothenburg University, Gothenburg, Sweden

^f^Wallenberg Centre for Molecular and Translational Medicine, Institute of Medicine, University of Gothenburg, Gothenburg, Sweden

^g^Clinical Trial Centre, Cardiovascular Research Foundation, New York, USA

**ADDRESS FOR CORRESPONDENCE:**

Bjorn Redfors, MD, PhD

Department of Cardiology,

Sahlgrenska University Hospital/S

Bruna Straket 16, 431 45 Gothenburg, Sweden.

Email: [bjorn.redfors@wlab.gu.se](mailto:mfg9004@med.cornell.edu)

+46 31-786 64 16

| Table S1 | Eligibility criteria | | **Page 2** |
| --- | --- | --- | --- |
| Table S2 | Study-related procedures/Schedule of events | | **Pages 3** |
| Table S3 | Periprocedural drugs and drugs at discharge | | **Pages 4-6** |
| Table S4 | Electrocardiogram-data | | **Pages 7-8** |
| Table S5 | Echocardiography data | | **Pages 9-10** |
| Table S6 | Biomarker data | | **Pages 11-12** |
| Figure S1 | Subgroup analysis for age | | **Page 13** |
| Supplemental Appendix 1 | STAMI echocardiography manual of procedure | | **See separate file** |
|  |  |  | |

**Table S1. Eligibility criteria.**

| **Inclusion criteria** |
| --- |
| 1. STEMI, TS, or other cardiac conditions with anticipated myocardial stunning, including but not limited to:    - Resuscitation after cardiac arrest;    - Cardiac surgery;    - Tachycardia-induced cardiac dysfunction;    - Pulmonary embolism;    - Acute heart failure or non-ischemic myocardial stunning. 2. Patients with STEMI: Primary PCI performed, and symptom onset must be within 6 hours of primary PCI. 3. Written informed consent. |
| **Exclusion criteria** |
| 1. Prior acute myocardial infarction or known pre-existing persistent regional myocardial dysfunction. 2. Expected inability to comply with the protocol. |

**Table S2. Study-related procedures/Schedule of events.**

| Schedule of events | Baseline/  Presentation | 24  ± 6h | 48  ±12h | 72  ±12h | 7 days  ± 48h | 14 days  ± 48h | 30 days  ± 48h |
| --- | --- | --- | --- | --- | --- | --- | --- |
| Informed consent* | X |  |  |  |  |  |  |
| Eligibility criteria | X |  |  |  |  |  |  |
| History and risk factors | X |  |  |  |  |  |  |
| QoL and chest pain questionnaires | X** |  |  |  |  |  |  |
| Recording of medications | X | X | X | X | X | X | X |
| Continuous telemetry monitoring# | X | X | X | X |  |  |  |
| Blood pressure and heart rate† | X | X | X | X | X | X | X |
| 12-Lead ECG & VCG | X | X | X | X | X | X | X |
| Echocardiography | X | X | X | X | X | X | X |
| Troponins and NT-proBNP | X | X | X | X | X | X | X |
| Serum creatinine, haemoglobin,  WBC and electrolytes‡ | X |  |  |  |  |  |  |
| Blood samples for biobanking | X | X | X | X | X | X | X |
| Outcomes$ |  |  |  |  |  |  | X |

*Because all examinations and activities are part of routine care after STEMI, TS or other conditions with stunning, the patient may be enrolled later in the course of the study, as long as echocardiography has been performed in accordance with the protocol (e.g. as standard of care).

**Collected by telephone or letter retrospectively if not available at presentation. #Continuously recorded throughout duration of the index hospitalization. †Blood pressure and heart rate will be recorded every 12 hours. ‡ Part of routine medical care for all patients with STEMI or TS. $By phone or visit.

QoL=quality of life; ECG = electrocardiography; VCG = vectorcardiography; WBC = white blood cell count.

**Table S3. Periprocedural drugs**

| **Periprocedural drugs** | **Anterior STEMI**  N = 41 | **Takotsubo**  N = 61 |
| --- | --- | --- |
| Morphine administered before arrival at lab | 23/41 (56.1%) | 10/61 (16.4%) |
| Nitroglycerin administered before arrival at lab | 24/41 (58.5%) | 24/61 (39.3%) |
| Oac administered before lab | 5/41 (12.2%) | 8/61 (13.1%) |
| Heparin administered before arrival at lab | 35/41 (85.4%) | 28/61 (45.9%) |
| Morphine administered at lab | 19/41 (46.3%) | 2/61 (3.3%) |
| Glycoprotein IIb/IIIa administered at lab | 2/41 (4.9%) | 0/61 (0.0%) |
| Beta blockers |  |  |
| Beta blocker administered acutely | 0/41 (0.0%) | 2/61 (3.3%) |
| On beta blocker since before | 6/41 (14.6%) | 12/61 (19.7%) |
| No beta blocker before lab | 35/41 (85.4%) | 47/61 (77.0%) |
| ACEi, ARB, or neprilysin inhibitor |  |  |
| ACEi, ARB or neprilysin inhibitor administered acutely | 1/41 (2.4%) | 1/61 (1.6%) |
| One ACEi, ARB or neprilysin inhibitor since before | 8/41 (19.5%) | 21/61 (34.4%) |
| No ACEi, ARB or neprilysin inhibitor before lab | 32/41 (78.0%) | 39/61 (63.9%) |
| Calcium channel inhibitor |  |  |
| On calcium channel inhibitor since before | 4/41 (9.8%) | 5/61 (8.2%) |
| No calcium channel inhibitor before lab | 37/41 (90.2%) | 56/61 (91.8%) |
| ASA |  |  |
| ASA administered at lab | 2/41 (4.9%) | 0/61 (0.0%) |
| ASA administered before arrival at lab | 36/41 (87.8%) | 45/61 (73.8%) |
| ASA administered first at hospital ward after lab | 2/41 (4.9%) | 0/61 (0.0%) |
| No ASA | 1/41 (2.4%) | 16/61 (26.2%) |
| P2Y12i |  |  |
| P2Y12 inhibitor administered at lab | 37/41 (90.2%) | 2/61 (3.3%) |
| P2Y12 inhibitor administered before arrival at lab | 1/41 (2.4%) | 1/61 (1.6%) |
| P2Y12 inhibitor administered first at hospital ward after lab | 1/41 (2.4%) | 0/61 (0.0%) |
| No P2Y12 inhibitor | 2/41 (4.9%) | 58/61 (95.1%) |
| Clopidogrel | 5/39 (12.8%) | 2/3 (66.7%) |
| Ticagrelor | 31/39 (79.5%) | 1/3 (33.3%) |
| Prasugrel | 3/39 (7.7%) | 0/3 (0.0%) |
| Cangrelor | 1/39 (2.6%) | 0/3 (0.0%) |
| Phenylephrine administered at lab | 1/41 (2.4%) | 3/61 (4.9%) |
| Noradrenaline administered at lab | 0/41 (0.0%) | 1/61 (1.6%) |
| Dobutamine administered at lab | 1/41 (2.4%) | 0/61 (0.0%) |
| Adrenaline administered at lab | 1/41 (2.4%) | 1/61 (1.6%) |
| Levosimendan administered after lab | 1/41 (2.4%) | 0/61 (0.0%) |
| Glycoprotein IIb/IIIa administered after lab | 2/41 (4.9%) | 0/61 (0.0%) |
| Phenylephrine administered after lab | 0/41 (0.0%) | 1/61 (1.6%) |
| Noradrenaline administered after lab | 1/41 (2.4%) | 3/61 (4.9%) |
| Milrinone administered after lab | 0/41 (0.0%) | 1/61 (1.6%) |
| Other vasopressor or inotropes administered after lab | 0/41 (0.0%) | 2/61 (3.3%) |

ASA = Acetylsalicylic acid; ACEi = Angiotensin-converting-enzyme inhibitor; ARB = Angiotensin receptor blocker; OAC = Oral anticoagulants; P2Y12i = P2Y12 inhibitor.

Data are shown as n (%).

**Table S4. Electrocardiogram data**

| **Variable** | **Anterior STEMI**  N = 41 | **Takotsubo**  N = 61 |
| --- | --- | --- |
| Heart rate (BPM) | 80.8 ± 18.4 | 97.3 ± 23.9 |
| ECG rhythm |  |  |
| Sinus Rhythm | 40/40 (100.0%) | 54/57 (94.7%) |
| Atrial Tachycardia | 0/40 (0.0%) | 2/57 (3.5%) |
| Junctional Rhythm | 0/40 (0.0%) | 1/57 (1.8%) |
| PR interval (ms) | 161.3 ± 23.4 | 162.5 ± 38.7 |
| QRS interval (ms) | 91.4 ± 16.3 | 90.7 ± 15.4 |
| Pathological Q qave | 12/41 (29.3%) | 20/61 (32.8%) |
| Fragmented QRS | 16/41 (39.0%) | 25/61 (41.0%) |
| cQT Interval (Bazett) | 449.0 ± 30.6 | 466.9 ± 53.4 |
| QT Interval | 392.9 ± 42.2 | 373.8 ± 52.5 |
| ECG axis | 32 (-1, 67) | 13 (-32, 55) |
| Bundle branch block |  |  |
| Left Bundle Branch Block | 1/41 (2.4%) | 0/61 (0.0%) |
| ST elevation | 40/41 (97.6%) | 37/61 (60.7%) |
| ST depression | 18/41 (43.9%) | 7/61 (11.5%) |
| Reciprocal ST depression | 10/18 (55.6%) | 3/7 (42.9%) |
| T wave |  |  |
| Hyperacute | 16/41 (39.0%) | 12/61 (19.7%) |
| Flatten | 2/41 (4.9%) | 19/61 (31.1%) |
| Biphasic | 5/41 (12.2%) | 3/61 (4.9%) |
| Negative | 11/41 (26.8%) | 24/61 (39.3%) |
| Normal | 16/41 (39.0%) | 27/61 (44.3%) |

BPM = Beats per minute; ECG = electrocardiogram; STEMI = ST-elevation myocardial infarction.

Data are shown as n (%) or mean ± standard deviation.

**Table S5. Echocardiography data**

| **Echocardiography parameters** | **Anterior STEMI**  N = 41 | **Takotsubo**  N = 61 |
| --- | --- | --- |
| **Akinesia (%)** |  |  |
| Day 0 | 36.5 [30.6, 42.4] | 44.8 [40.0, 49.5] |
| Day 1 | 35.4 [29.8, 41.0] | 41.4 [36.7, 45.9] |
| Day 2 | 28.2 [22.1, 34.4] | 34.1 [29.5, 38.7] |
| Day 3 | 17.5 [11.3, 23.7] | 26.7 [22.0, 31.3] |
| Day 7 | 12.1 [5.4, 18.4] | 4.3 [0.0, 9.3] |
| Day 14 | 2.6 [0.0, 10.2] | 0.0 [0.0, 0.0] |
| Day 30 | 0.6 [0.0, 8.1] | 0.0 [0.0, 0.0] |
| **Day 30 recovery achieved (%)** |  |  |
| Day 1 | 3.2 [-14.9, 18.4] | 7.6 [-4.8, 18.5] |
| Day 2 | 23.9 [6.0, 40.3] | 23.9 [12.8, 33.9] |
| Day 3 | 54.7 [38.3, 72.0] | 40.4 [30.1, 50.1] |
| Day 7 | 70.5 [53.6, 90.0] | 90.3 [79.7, 100.0] |
| Day 14 | 97.7 [77.2, 117.4] | 100.0 [100.0, 100.0] |
| **Patients fully recovered (%)** |  |  |
| Day 0 | 1.9 [0.6, 5.7] | 0.3 [0.1, 1.0] |
| Day 1 | 2.3 [0.7, 6.2] | 0.5 [0.1, 1.6] |
| Day 2 | 5.6 [2.1, 12.2] | 1.7 [0.6, 4.1] |
| Day 3 | 16.3 [8.5, 27.2] | 4.8 [2.1, 9.6] |
| Day 7 | 24.9 [14.6, 39.1] | 39.3 [27.5, 51.9] |
| Day 14 | 44.7 [28.5, 61.9] | 77.7 [63.2, 88.3] |
| Day 30 | 49.1 [32.6, 66.8] | 99.8 [93.3, 100.0] |
| **LVEF (%)** |  |  |
| Day 0 | 45.2 (41.1, 49.3) | 39.2 (35.9, 42.5) |
| Day 1 | 44.8 (40.9, 48.7) | 40 (36.7, 43.2) |
| Day 2 | 46.7 (42.6, 50.8) | 45.6 (42.6, 48.7) |
| Day 3 | 46.5 (42.3, 50.6) | 48.7 (45.4, 51.9) |
| Day 7 | 50.9 (46.6, 55.2) | 53.2 (50, 56.3) |
| Day 14 | 54.2 (50.1, 58.4) | 59.2 (55.9, 62.5) |
| Day 30 | 54.7 (50.4, 59.1) | 61.5 (58.3, 64.8) |
| **GLS (%)** |  |  |
| Day 0 | -11.8 (-13.1, -10.4) | -9.5 (-10.6, -8.4) |
| Day 1 | -11.1 (-12.4, -9.8) | -10.6 (-11.6, -9.5) |
| Day 2 | -11.4 (-12.7, -10) | -11.4 (-12.4, -10.3) |
| Day 3 | -11.8 (-13.3, -10.4) | -12.2 (-13.3, -11.2) |
| Day 7 | -13.9 (-15.4, -12.4) | -13.4 (-14.5, -12.4) |
| Day 14 | -14.7 (-16.1, -13.3) | -16.5 (-17.6, -15.3) |
| Day 30 | -16 (-17.5, -14.6) | -17.5 (-18.6, -16.3) |
| **TAPSE (mm)** |  |  |
| Day 0 | 23.8 (21.9, 25.6) | 17.1 (15.8, 18.4) |
| Day 1 | 22.6 (20.8, 24.5) | 18.8 (17.6, 20.1) |
| Day 2 | 22.4 (20.6, 24.3) | 20.5 (19.2, 21.8) |
| Day 3 | 21.8 (19.9, 23.8) | 20.8 (19.5, 22) |
| Day 7 | 22.5 (20.5, 24.5) | 21.8 (20.5, 23.1) |
| Day 14 | 23.8 (21.8, 25.9) | 22.7 (21.3, 24) |
| Day 30 | 22.8 (20.8, 24.9) | 22.5 (21.2, 23.8) |
| **WMSI** |  |  |
| Day 0 | 1.9 [1.7, 2.0] | 2.1 [2.0, 2.2] |
| Day 1 | 1.8 [1.7, 1.9] | 2.0 [1.9, 2.1] |
| Day 2 | 1.7 [1.6, 1.8] | 1.8 [1.7, 1.9] |
| Day 3 | 1.5 [1.4, 1.7] | 1.7 [1.6, 1.8] |
| Day 7 | 1.4 [1.3, 1.5] | 1.3 [1.2, 1.4] |
| Day 14 | 1.2 [1.0, 1.3] | 1.0 [1.0, 1.0] |
| Day 30 | 1.1 [1.0, 1.2] | 1.0 [1.0, 1.0] |

Data is shown as median (95% credible interval) for Akinesia, Day 30 recovery achieved, Patients fully recovered, and WMSI or as mean (95% confidence interval) for LVEF, GLS, and TAPSE. LVEF = left ventricular ejection fraction; GLS = global longitudinal strain; TAPSE = tricuspid annular plane systolic excursion; WMSI = wall motion score index.

**Table S6. Biomarker data**

| **Biomarkers** | **Anterior STEMI**  N = 41 | **Takotsubo**  N = 61 |
| --- | --- | --- |
| **Troponin-I (ng/L)** |  |  |
| Day 0 | 1613.2 (793.3, 3280.4) | 1381.5 (918.2, 2078.5) |
| Day 1 | 20974.9 (10259, 42883.9) | 1499.8 (986.5, 2280) |
| Day 2 | 11093 (5339.7, 23045.4) | 706.2 (459.7, 1084.7) |
| Day 3 | 5642.4 (2672.5, 11912.5) | 324.4 (209.2, 502.9) |
| Day 7 | 283.5 (134.8, 596.2) | 45.7 (29.6, 70.7) |
| Day 14 | 35.1 (16.8, 73.1) | 15.6 (10, 24.4) |
| Day 30 | 9.8 (4.7, 20.7) | 7.9 (5.1, 12.3) |
| **Troponin-T (ng/L)** |  |  |
| Day 0 | 482.2 (266.1, 873.9) | 465.3 (337, 642.3) |
| Day 1 | 1787.1 (999.7, 3194.8) | 431.4 (314.1, 592.7) |
| Day 2 | 1497 (824.4, 2718.4) | 221.7 (161.1, 305.1) |
| Day 3 | 1337.1 (734, 2436) | 184.8 (132.6, 257.6) |
| Day 7 | 227.7 (124.3, 417) | 48.5 (35.4, 66.4) |
| Day 14 | 32.5 (17.7, 59.6) | 22.3 (16.3, 30.4) |
| Day 30 | 17.2 (9.4, 31.5) | 12.5 (9.1, 17.1) |
| **Troponin-I/Troponin-T-ratio** |  |  |
| Day 0 | 3.5 (2.1, 5.9) | 3.9 (2.9, 5.1) |
| Day 1 | 12.3 (7.6, 20) | 5.4 (4.2, 6.8) |
| Day 2 | 7.7 (4.7, 12.5) | 4 (3.2, 5.2) |
| Day 3 | 4.5 (2.7, 7.2) | 2.6 (2, 3.3) |
| Day 7 | 1.7 (1.1, 2.9) | 1.1 (0.9, 1.5) |
| Day 14 | 1.4 (0.9, 2.3) | 0.9 (0.7, 1.2) |
| Day 30 | 0.7 (0.5, 1.2) | 0.8 (0.6, 1) |
| **NT-proBNP (ng/L)** |  |  |
| Day 0 | 451.2 (277.6, 733.2) | 997.2 (690.7, 1439.8) |
| Day 1 | 3059.5 (1943.1, 4817.5) | 4543.1 (3184, 6482.3) |
| Day 2 | 2484.5 (1561.7, 3952.7) | 4530.2 (3164, 6486.4) |
| Day 3 | 1942.8 (1208.8, 3122.4) | 2880 (1978.8, 4191.7) |
| Day 7 | 1409.5 (878.7, 2260.8) | 1506.1 (1055.2, 2149.8) |
| Day 14 | 1297.8 (812, 2074.2) | 1731.2 (1202.5, 2492.2) |
| Day 30 | 817.4 (509.4, 1311.6) | 559.3 (388.1, 806.1) |
| **NT-proBNP/Troponin-T-ratio** |  |  |
| Day 0 | 0.9 (0.4, 2) | 1.6 (0.9, 2.9) |
| Day 1 | 1.7 (0.8, 3.6) | 12.1 (7.2, 20.5) |
| Day 2 | 1.8 (0.9, 3.8) | 21.5 (12.7, 36.4) |
| Day 3 | 1.6 (0.8, 3.4) | 18.4 (10.4, 32.5) |
| Day 7 | 9.1 (4.2, 19.7) | 31.3 (18.5, 52.9) |
| Day 14 | 46.2 (21.9, 97.6) | 92.1 (54.5, 155.8) |
| Day 30 | 54.9 (26, 115.9) | 47.5 (28.4, 79.2) |
| **NT-proBNP/Troponin-I-ratio** |  |  |
| Day 0 | 0.3 (0.1, 0.5) | 0.7 (0.4, 1.2) |
| Day 1 | 0.1 (0.1, 0.3) | 2.7 (1.7, 4.4) |
| Day 2 | 0.2 (0.1, 0.4) | 5.9 (3.6, 9.5) |
| Day 3 | 0.3 (0.2, 0.7) | 7.9 (4.8, 12.9) |
| Day 7 | 4.7 (2.3, 9.4) | 32 (19.9, 51.5) |
| Day 14 | 36.6 (18.3, 72.9) | 106.5 (65.6, 172.9) |
| Day 30 | 79.5 (39.6, 159.6) | 68.2 (41.9, 110.9) |

Data is shown as mean (95% confidence interval).

STEMI = ST-elevation myocardial infarction.

**Supplemental Figure 1.**


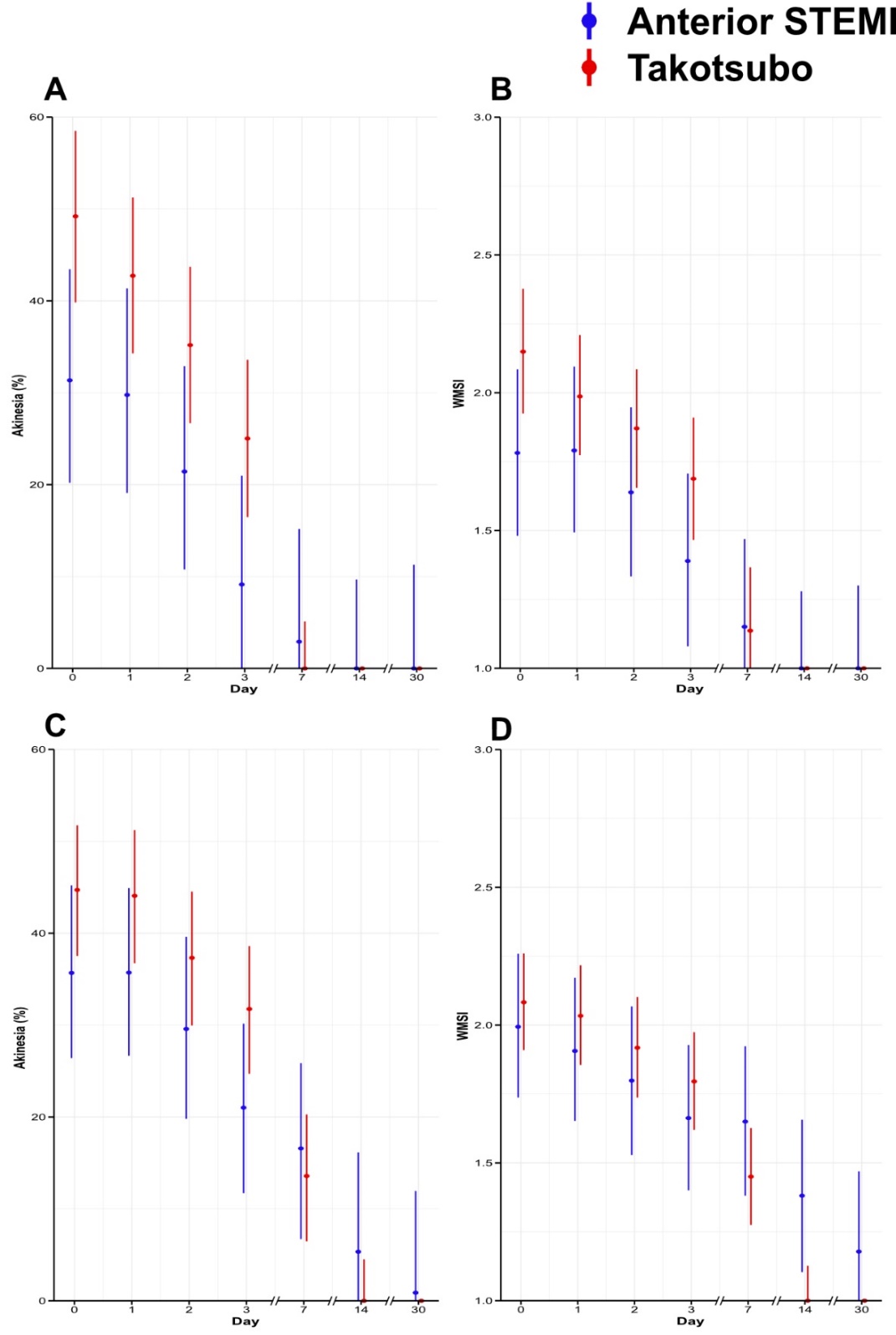


**Supplemental Figure 1.** Age sub-group analysis. Predicted medians of Percentage akinesia and WMSI in patients below the age of 75 (**A-B**) and patients ≥75 years of age (**C-D**).

WMSI = Wall motion score index.
