# Supplementary material for: Prospective comparison of temporal changes in myocardial function in women with Takotsubo versus anterior STEMI
Source: Clin Res Cardiol. 2025 Mar 20;114(12):1705–17. doi: 10.1007/s00392-025-02633-4 (PMC12708765; doi:10.1007/s00392-025-02633-4)
Supplement: Supplementary file 2 — Supplementary file2 (DOCX 6729 KB) [file 392_2025_2633_MOESM2_ESM.docx]

**Manual of procedure - STAMI**

**Echocardiography guidelines**

Transthoracic echocardiography (TTE) with Doppler is a non-invasive method to investigate the systolic and diastolic function of the heart, the morphology and function of the heart valves and to assess and measure intra cardiac flows and pressure, by using a transducer with piezo-electrical crystals. These are stimulated by an electrical pulse to release sound waves. Most of the waves absorbs by the body but those that interfaces between different tissue density are reflected. The transducer detects the reflecting sound waves, processes the information and represent it as an image. By using the Doppler-method it is possible to measure and estimate intra cardiac velocities and pressure and to evaluate blood flow. There are four different kinds of Doppler used in echocardiography:

- Color flow Doppler (CFD): CFD measures velocity and direction of the blood flow and present it as a color pattern into a region of interest of the image. Usually, flow towards the transducer is red and blood away from the transducer is blue.
- Continuous-wave Doppler (CWD): Measures high velocities along the entire ultrasound beam. It can not localize where along the ultrasound beam the highest velocity is.
- Pulsed-wave Doppler (PWD): Measures blood flow velocities at a specified depth. The disadvantages with PWD is that it can not measure high velocities.
- Tissue Doppler imaging (TDI): Measures the velocity of the myocardium throughout the cardiac cycle.

This guideline outlines a structured approach to image acquisition and a standardized measurement protocol for performing a standard TTE in Stunning in Acute Myocardial Infarction and Takotsubo syndrome (STAMI). STAMI aims to assess myocardial stunning resolution in patients with STEMI or Takotsubo cardiomyopathy.

**Quality aspects**

It is important that the echocardiographer understand how to optimize the images. It is also important that the echocardiographer understand the basic ultrasound physics. Higher scanning frequencies gives a higher image resolution, but with limited depth penetration, whereas lower scanning frequencies can penetrate deeper into the body but provide lower image resolution. To be able to perform measurements the quality of the images has to be high. If the image quality is too low measurements is not recommended.

**Preparations**

The patient should be informed that echocardiography shall be performed. The echocardiographer should ensure patient privacy at all times and should allow the patient to be positioned comfortably in the left lateral decubitus with their left arm raised or left hand under their head. An ECG should be attached to ensure good tracing facilities at the acquisition of complete loops.

TTE should be performed in a room with the right facilities to obtain the highest image quality. If the patient can not leave the ward TTE can be performed bedside.

**Identifying information**

The images acquired should be clearly labelled with patient identification, including the following:

- Patient name
- Personal identification number

**Equipment**

The equipment used are:

- GE Vivid E95
- E9

**Echocardiography protocol in baseline**

| 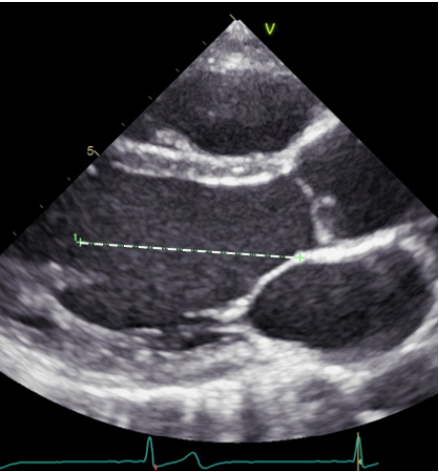 | **Optimal PLAX**  Parasternal long axis view.  Scan depth is set to optimize the PLAX with about 1 cm beyond the pericardium remaining within the image.  The long axis should be horizontal. It is important to find the image were the long axis has the greatest diameter.  In case of poor image quality   - Lean the patient more forward - Raise the patient’s left arm up next to the pillow - Lower the transducer frequency |
| --- | --- |
| 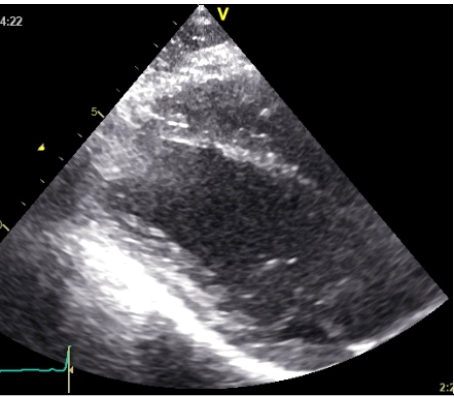 | **Inaccurate PLAX oblique, tipped**  This image is not optimal. The left ventricle is not horizontal.  Move the transducer closer to sternum, closer to the patient’s head. Lean the patient more forward. |
| 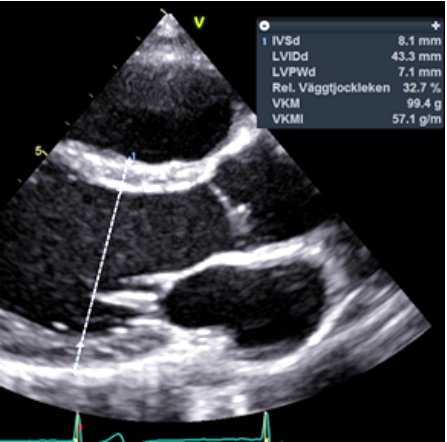 | **How to measure septum, LV-diameter and the posterior wall**  Measurement of septum, LV-diameter and posterior wall is done in the image where the LV is horizontal. The measurements are made perpendicular to the LV long axis between the leaflet of the mitral valve and the papillary muscle.  The measurements are made in end-diastole and end-systole. Trabeculae and corda should not be included. |
| 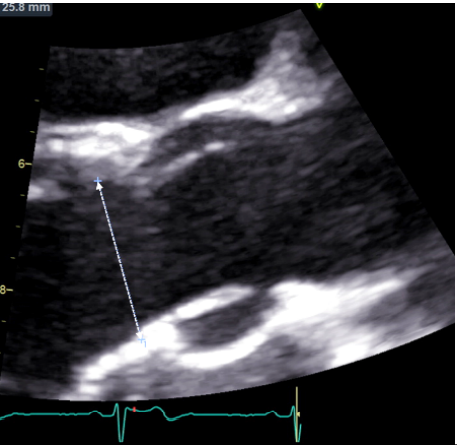 | **LVOT zoom**  LVOT - left ventricular outflow tract. Two cusps are visible; the right coronary cusp is positioned anteriorly and extends from the ventricular septum. The more posterior cusp may be either the non-coronary cusp or the left coronary cusp. When measure the LVOT use the frame providing the largest LVOT, usually in early systole, as inner diameter (trailing to leading edge). |
| 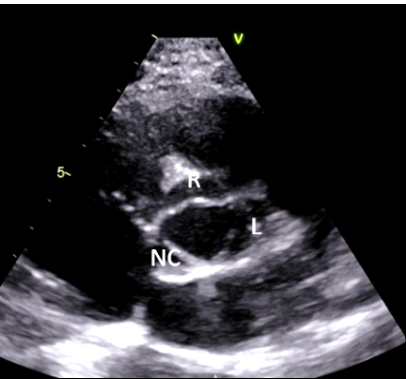 | **PSAX aortic valve**  Rotate the transducer about 90^o^ to visualize the aortic short axis view. An optimal PSAX of the aortic valve demonstrate the aortic valve in the center of the image. The depth is set to around 1 cm beyond the left atrial posterior wall.  This is a normal tricuspid aortic valve.  NC= Noncoronary cusp  R= Right coronary cusp  L= Left coronary cusp |
| 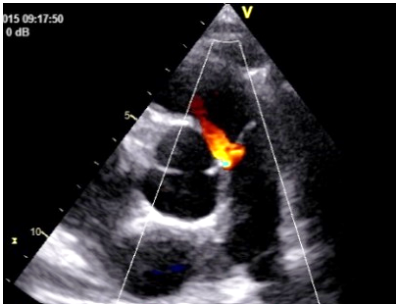 | **Pulmonary artery color Doppler**  Place the color Doppler box over the pulmonary artery to assess pulmonary regurgitation and any abnormal flow. |
| 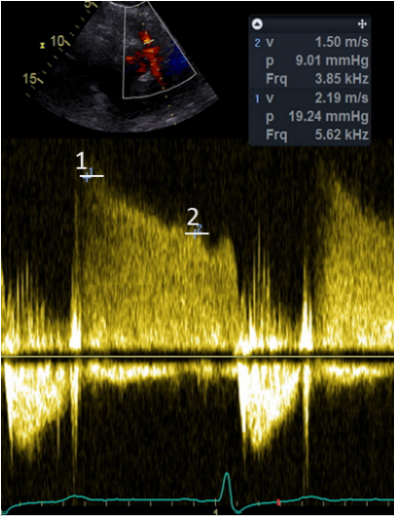 | **Pulmonary artery CWD**  Place CWD in the start of the pulmonary regurgitation. The regurgitation should be parallel to the Doppler cursor line. |
| 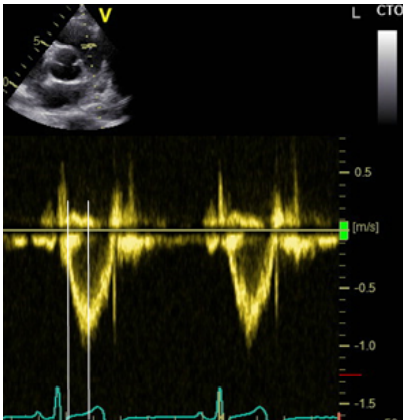 | **Pulmonary artery PWD**  Place the sample volume just above the pulmonary artery valve. The flow should be aligned to the cursor line. |
| 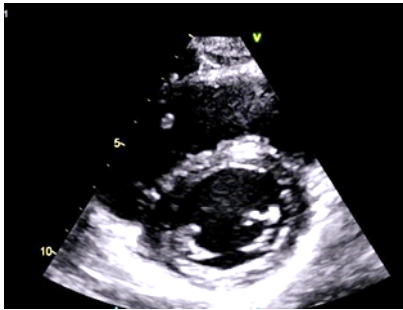 | **PSAX Basal LV**  The LV should be circular.  In case of oval LV, place the transducer closer to sternum.  The image aims to view the basal segments and to visualize myocardial mobility. The mitral valve should not be visible.  In all PSAX views the right ventricle should be viewed. |
| 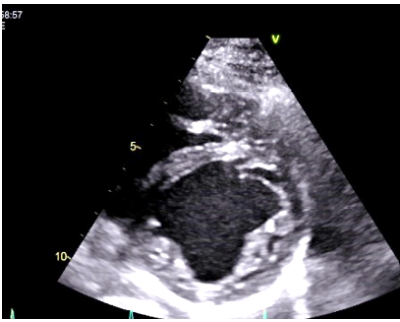 | **PSAX mid LV**  The PSAX of the middle of the LV aims to view the mid parts of the LV, its mobility and the papillary muscle. |
| 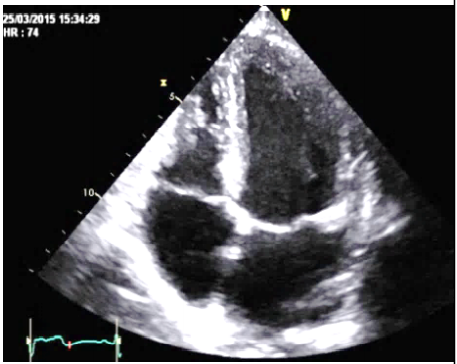 | **Apical four chamber view (A4C), optimal**  In an optimal A4C all the four chambers are seen. The LV should be as long as possible. Septum and apex should be in the center of the image. |
| 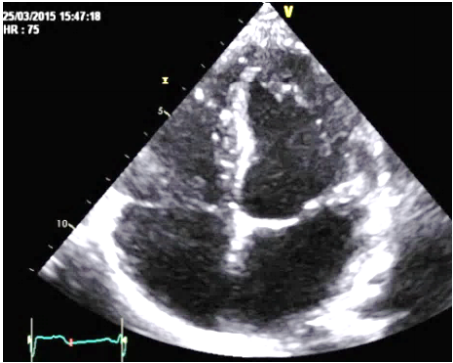 | **Foreshortened A4C**  This is a common incorrect A4C. Apex is foreshortened and round, in systole apex moves towards the mitral valve. Usually, the right ventricle seems to be bigger than reality.  To find a more correct view:   - Move the transducer more dorsal. - Move the transducer towards the patient’s feet. Ask the patient to inhale. - Lean the patient more forward. |
| 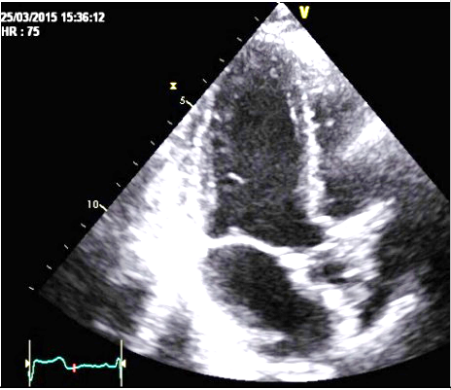 | **Apical three chamber, ALAX (A3C)**  This image visualizes the same segments as PLAX. Place the transducer in the A4C view, rotate it about 180^◦^. The transducer notch should be directed towards the patient’s right shoulder. The LV should be as long as LV in A4C. |
| 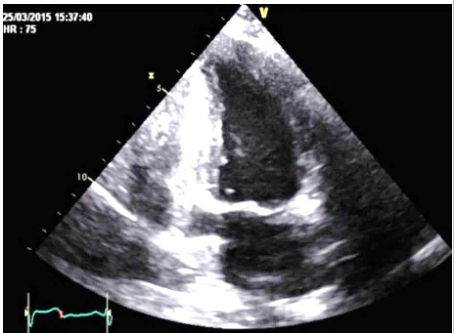 | **Apical two chamber (A2C)**  Start from an optimal A3C. Rotate the transducer clockwise until the aorta disappears from the view, about 90^◦^. The LV should be as long as LV in A4C. Usually the inferior papillary muscle is seen. |
| 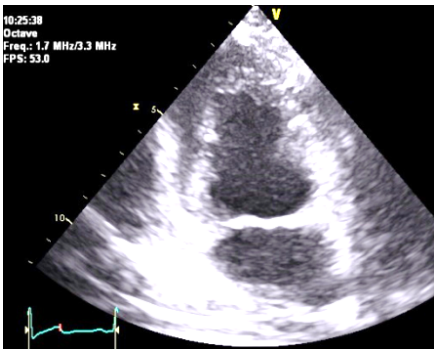 | **Foreshortened A2C**  This is a common incorrect A2C. Apex is foreshortened and round, in systole apex moves towards the mitral valve.  To find a more correct view:   - Ask the patient to inhale. - Move the transducer backwards or an interstitial further down. |
| 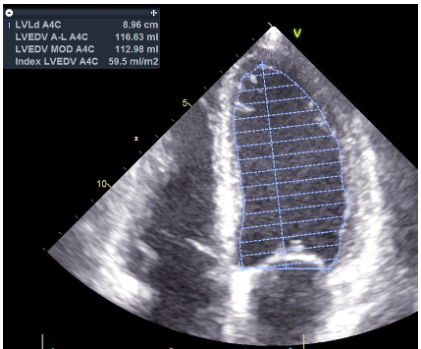 | **How to measure LV volume using Simpson**  A4C, diastole  Use the frame where the mitral valve has closed and the LV is the greatest. The image shows some intraventricular bands, these are included in the LV cavity. Even the papillary muscles are included in the LV cavity.  If ≥25% of LV wall is not visible, Simpson is not recommended. |
| 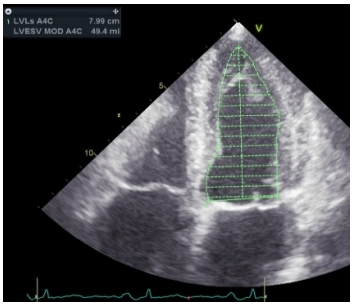 | **How to measure LV volume using Simpson**  A4C, systole  Use the frame where LV is the smallest.  The image shows some trabeculae in apex that are included in the LV cavity. |
| 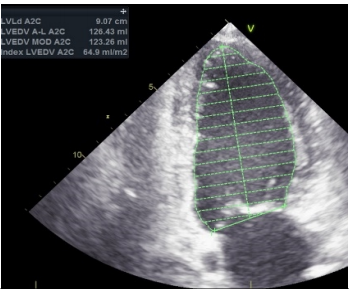 | **How to measure LV volume using Simpson**  A2C, diastole  In an optimal image the A2C is as long as A4C. |
| 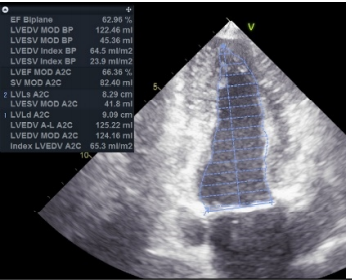 | **How to measure LV volume using Simpson**  A2C, systole |
| 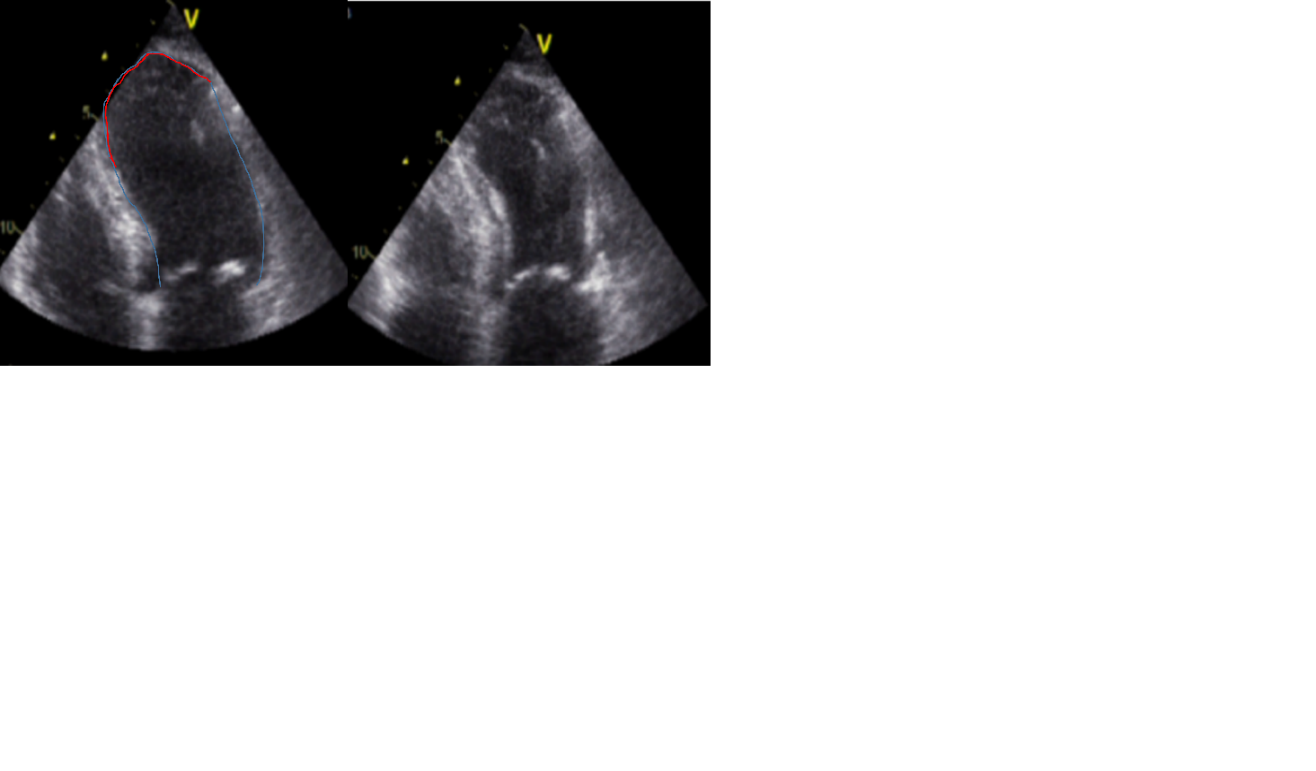 | **Estimation of akinesia and hypokinesia**  Use the calculation tool “area”. Trace the endocardium in diastole the same way as Simpson method. Trace the region of akinesia. Then trace the area of both akinesia and hypokinesia. This is made in both A4C and A2C. |


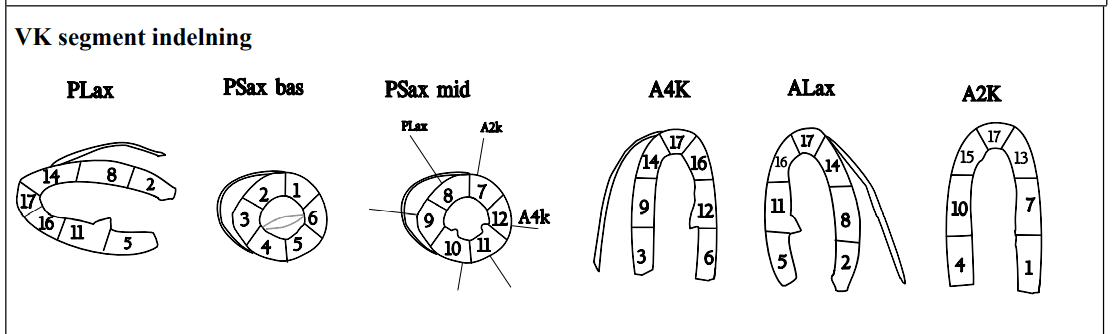


| 1. Basal anterior, LAD | 7. Mid anterior, LAD | 13. Apical anterior, LAD |
| --- | --- | --- |
| 2. Basal anteroseptal, LAD | 8. Mid anteroseptal, LAD | 14. Apical anteroseptal, LAD |
| 3. Basal septal, RCA | 9. Mid septal, LAD | 15. Apical septal, LAD |
| 4. Basal Inferior, RCA | 10. Mid inferior, RCA | 16. Apical inferolateral, LAD |
| 5. Basal inferolateral, LCX | 11. Mid inferolateral, LCX | 17. Apical apex, LAD |
| 6. Basal lateral, LCX | 12. Mid lateral, LCX |  |

| 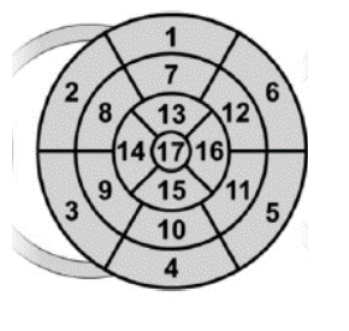 | **Bullseye**  LV mobility is assessed with the same 17 segment model as scintigraphy and CT. The regional mobility is based on LV thickening and is graded as: normal, hypokinesia, akinesia and dyskinesia. |
| --- | --- |
| 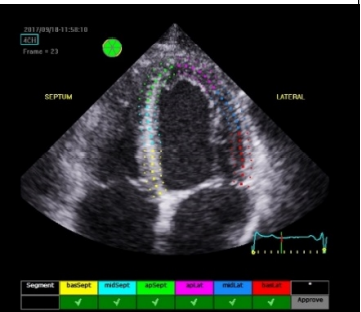 | **LV strain A4C**  To be able to perform LV strain all the myocardium has to be visualized. Framerate has to be at least 40-50 frames per second (FPS). A4C, A3C and A2C all have to be optimal.  Trace along the endocardium (same as Simpson method). Narrow the region of interest (ROI), the pericardium should not be included in the ROI. Trabeculae and papillary muscles should not be included in the ROI. |
| 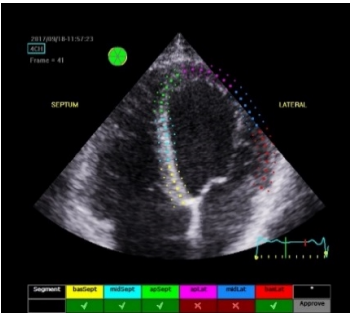 | **Incorrect LV strain**  Apical and mid parts of LV are not fully visualized in the image. These segments cannot be analyzed (red cross). |
| 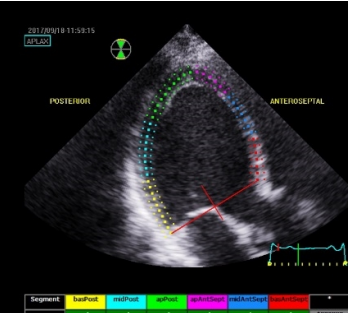 | **LV strain A3C**  LVOT should not be included in ROI. Use aortic valve closure (AVC) from spectral-Doppler and use end-systolic strain.  Strain in the basal and mid parts of the heart covariates with tissue Doppler. Normally, the strain values are greatest in apex. |
| 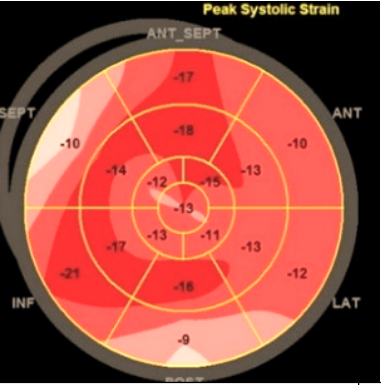 | **LV strain LAD stenosis**  This image shows an example of LV strain in a patient with LAD stenosis. Strain is reduced in the apical, lateral and anterior segments.  The strain curves are shown below. |
| 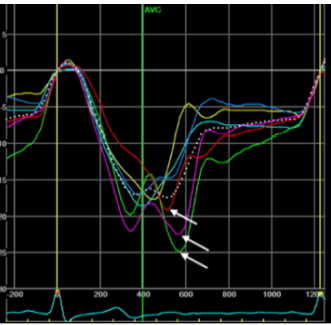 | **LV strain LAD stenosis**  The curves are stretched in early systole (positive strain). After AVC the post systolic contraction is shown in the apical and anterior segments (white arrows). This, together with reduced strain in the area supplied by LAD are characteristic for ischemia. |
| 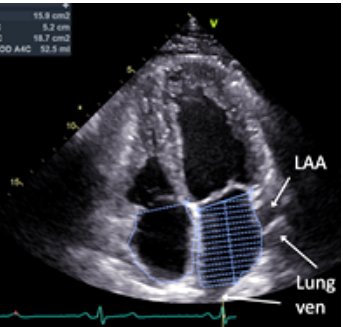 | **Atrium A4C**  Focus on the atriums in a 4C. The left atrium should be as big as possible without decreasing right atrium.  Measure the atriums, just before mitral valve opening. |
| 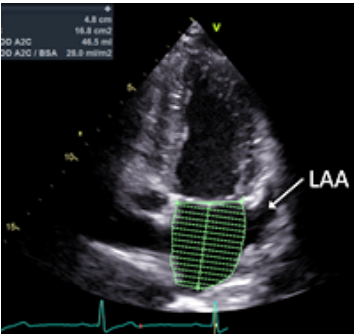 | **Atrium A2C**  In A2C focus on the left atrium. |
| 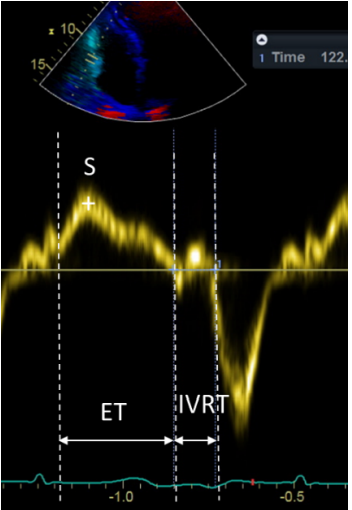 | **TDI Right Wall**  TDI in the right ventricular (RV) free wall is used to assess RV mobility and RV systolic pressure. The cursor should be parallel to the RV free wall.  S = systolic velocity  ET = ejection time  IVRT = Isovolumetric relaxation time |
| 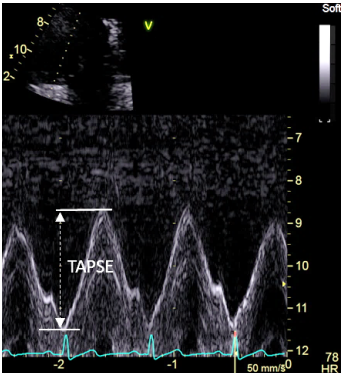 | **Tricuspid annulus plane systolic excursion (TAPSE)**  Can be used to assess RV mobility.  Use a standard A4C, place the M-mode cursor parallel to RV free wall. Use zoom to improve measurements accuracy. Measure along the same echo throughout systole. |

**Doppler**

Adjust gain. If color flow is seen in the tissue, gain is to high. When doing continuous or pulsed Doppler the background should be black. Adjust the scale after the velocity.

| 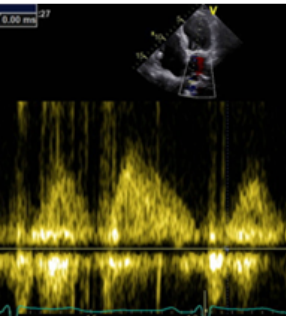 | **PWD lung vein**  Use color Doppler to detect the lung vein closest to atrial septum, the right upper lung vein.  Place the sample volume about 1 cm in the lung vein. |
| --- | --- |
| 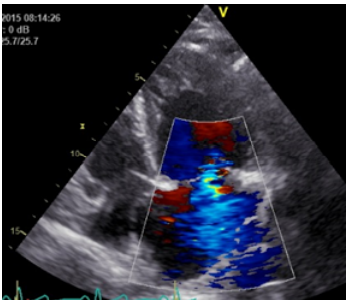 | **CFD mitral valve**  Use a narrow color box, frame rate (FR) is preferably more than 20 frames/second (FPS). The wider color box the less FR. The length of the color box does not affect FR. |
| 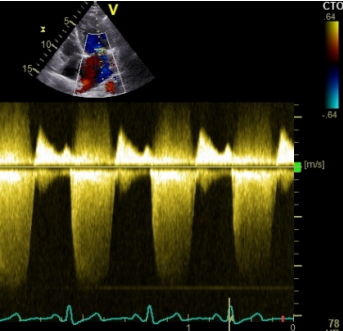 | **CWD mitral valve**  Adjust the angle. The flow registration should have the smallest possible angular error.  Scale should be high, about 4-6 m/s under the baseline and 2 m/s over the baseline. |
| 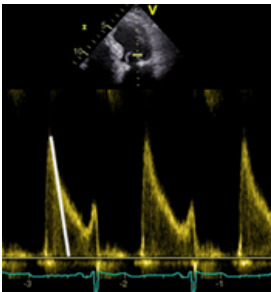 | **PWD Mitral valve**  Place the sample volume on the top of the Mitral leaflet (about 1-3 mm). |
| 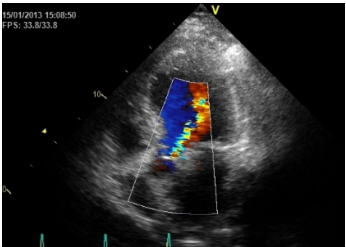 | **CFD aortic valve**  Angle the transducer to visualize the aortic valve and ascending aorta. The color box should cover the aortic valve and aortic regurgitation (AR). |
| 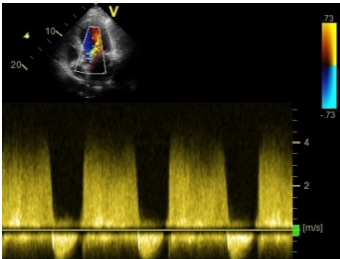 | **CWD aortic valve**  Place the CWD aligned to the color flow. |
| 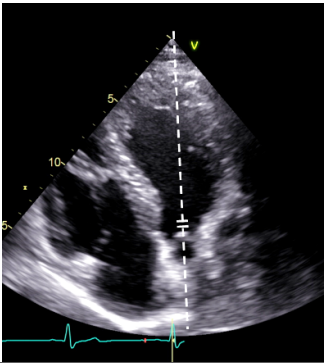 | **PWD LVOT**  The cursor should be aligned to LVOT.  Place the sample volume in LVOT about ½ cm from the aortic valve. |
| 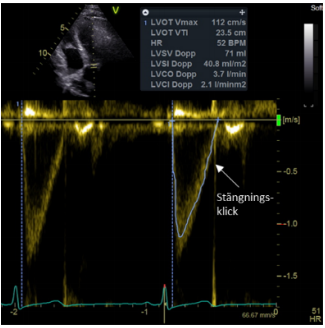 | **PWD LVOT, optimal**  Place the sample volume on the immediate LV side of the aortic valve at the same level as the LVOT diameter is measured. |
| **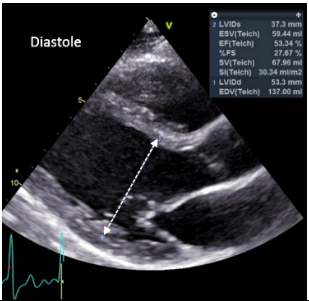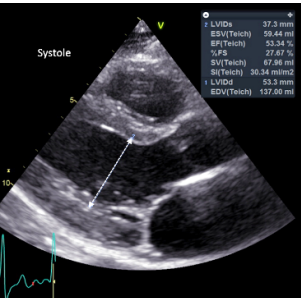** | **Validation of reasonableness in measurements of stroke volume**  There is a relationship between LV size, EF and stroke volume. Measure the diameters in both diastole and systole, make sure EF seems reasonble according to evaluated EF. Stroke volume using Teichholz should be approximately the same as Doppler. |

**Tricuspid Doppler**

Place the transducer parallel to the tricuspid regurgitation (TR). Mostly a modified right ventricular (RV) view is the best to estimate TI.

| 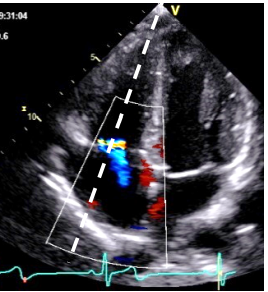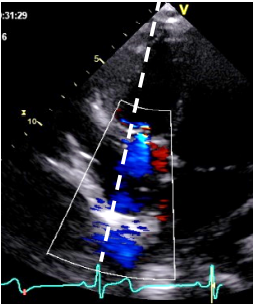 | **CFD Tricuspid valve**  The image to the right is the modified RV. Use CFD to detect tricuspid regurgitation (TR). Place the CWD aligned to TR. |
| --- | --- |
| 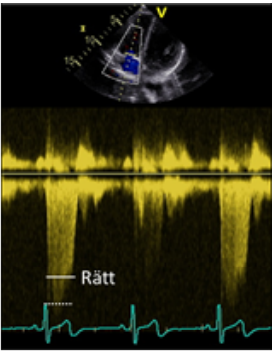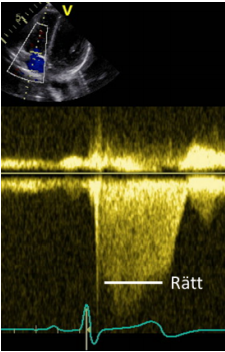 | **CWD Tricuspid valve**  To estimate TR velocity, use a high sweep speed to differentiate between true velocity and artefacts. |
| 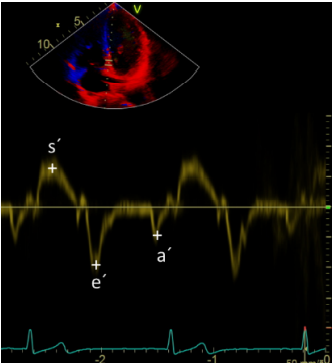 | **TDI**  Tissue Doppler is made in the lateral, septal and RV free wall. Place the cursor aligned to the wall. The sample volume is placed in the valve annulus during systole. |
| 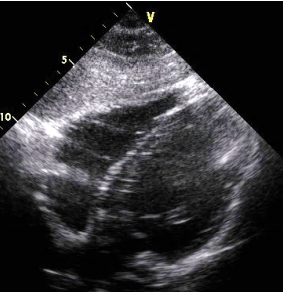 | **Subcostal four chamber**  Subcostal four chamber view.  Place the transducer with the notch towards you (patient lying on his back). |
| 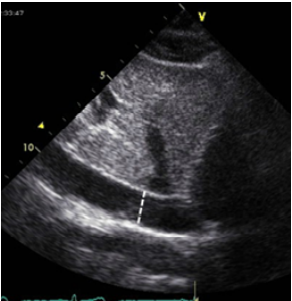 | **Subcostal vena cava inferior**  This image shows a long axis view of inferior vena cava (IVC) and right atrium.  Do the “sniff-test” and register a cine loop with at least three beats.  IVC diameter is measured perpendicular to the long axis view, about 1 cm from the right atrium junction. |

**Sonographer training**

All echocardiographic examinations will be conducted by highly experienced biomedical scientist (BMA) and physicians. All images will be digitally stored on a dedicated workstation for both clinical and scientific use. All new sonographers or physicians in the STAMI study will acquire formal training in the study protocol and will have access to hands-on training under the supervision by BMA and Cardiologists. All new participants/sonographers will be required to complete a preintervention assessment, a teaching intervention, and a postintervention assessment as per protocol.

**Preintervention assessment**

Participants will attend a briefing session describing the introductory modules of study and method protocol.

**Training intervention**

After completing the initial assessment, each participant attended a single 1-hour training session. The 1-hour training session includes the provision of an echocardiography training manual, 45 minutes of lecture-format delivery of the guide’s contents, and 15 minutes of interactive example image analysis and group discussion.

**Postintervention reassessment**

Similar to the preintervention assessment, the postintervention reassessment will be conducted with the revision of method protocol. 10 or more images will be randomly selected, which participants shall analyze.

**Inter- and intra-variability**

Inter and intra variability studies are going to be made regularly between Biomedical scientists and Cardiologists performing the echocardiographic measurements.

**Echocardiographic measurements**

| **Echocardiographic measurement** | **Abbreviation** | **Range** |
| --- | --- | --- |
| Left ventricular end-diastolic diameter (mm) | LVEDD |  |
| Left ventricular end-systolic diameter (mm) | LVESD |  |
| Left ventricular end-diastolic volume (ml) | EDV |  |
| Left ventricular end-systolic volume (ml) | ESV |  |
| Left ventricular septal wall diameter (mm) | LV_septum |  |
| Left ventricular poster wall diameter (mm) | LV_posterior |  |
| Left atrial area (cm^2^) | LAA |  |
| Left atrial volume (ml) | LAV |  |
| Left atrial diameter (mm) | LAD |  |
| Left ventricular ejection fraction visual (%) | LVEFv |  |
| Left ventricular ejection fraction Simpson method (%) | LVEFs |  |
| Aortic regurgitation | AI | None =0  Very small=0,5  Small=1  Moderate=2  Severe= 3 |
| Mitral regurgitation | MI | None =0  Very small=0,5  Small=1  Moderate=2  Severe= 3None =0 |
| Pulmonary regurgitation | PI | None =0  Very small=0,5  Small=1  Moderate=2  Severe= 3 |
| Tricuspid regurgitation | TI | None =0  Very small=0,5  Small=1  Moderate=2  Severe= 3 |
| Gradient over tricuspid valve (mmHg) | TI_gradient |  |
| Central venous pressure (mmHg) | CVT |  |
| Pulmonary arterial systolic pressure (mmHg9 | RA_pressure |  |
| Peak E (m/s) | E |  |
| Peak A (m/s) | A |  |
| E/A | E/A |  |
| Peak S (m/s) | S |  |
| Peak e' (m/s) | e' |  |
| Peak a' (m/s) | a' |  |
| Peak s' (m/s) | s' |  |
| E/é | E/e |  |
| S/D | S/D |  |
| E-wave deceleration time (ms) | Dec_time |  |
| Tricuspid annular plane systolic excursion (mm) | TAPSE |  |
| Aortic valve transvalvular max pressure gradient (mmHg) | AV_maxgradient |  |
| Aortic valve transvalvular mean pressure gradient (mmHg) | AV_medelgradient |  |
| Velocity time integral left ventricular outflow tract (cm) | VTI_LVOT |  |
| Left ventricular outflow tract diameter (mm) | LVOTd |  |
| Stroke volume (mm) | Strokevolume |  |
| Heartrate | Heartrate |  |
| Cardiac output (l/min) | CO |  |
| Aortic valve area (cm^2^) | AV_area |  |
| Left ventricular morphology comments | LV_morphology_comments |  |
| Apical ballooning | Apical_ballooning | No=0  Yes=1 |
| Midventricular ballooning | Midventricular | No=0  Yes=1 |
| Ventricular ballooning inverted forms | Inverted_forms | No=0  Yes=1 |
| Biventricular ballooning | Biventricular_ballooning | No=0  Yes=1 |
| Thrombus | Trombus | No=0  Yes=1 |
| Cardiac rupture | Cardiac_ruture | No=0  Yes=1 |
| Left ventricular outflow tract obstruction | LVOTO | No=0  Yes=1 |
| Systolic anterior motion of the mitral leaflet | SAM | No=0  Yes=1 |
| Global longitudinal strain left ventricle (%) | GLS |  |
| End-diastolic endocardial length of akinetic segment in 4 chamber view (mm) | Akinesia_length_4C |  |
| End-diastolic endocardial length of hypokinetic segment in 4 chamber view (mm) | Hypokinesi_and_akinesi_lenght_4C |  |
| End-diastolic endocardial length of left ventricle in 4 chamber view (mm) | Total_lenght_4C |  |
| End-diastolic endocardial length of akinetic segment in 2 chamber view (mm) | Akinesia_length_2C |  |
| End-diastolic endocardial length of hypokinetic segment in 2 chamber view (mm) | Hypokinesi_and_akinesi_lenght_2C |  |
| End-diastolic endocardial length of left ventricle in 2 chamber view (mm) | Total_lenght_2C |  |
| Contractile function in segment 1 (mm) | Seg_1 | Normal= 1  Hypokinesia =2  Akinesia=3  Dyskinesia =4 |
| Contractile function in segment 2 (mm) | Seg_2 | Normal= 1  Hypokinesia =2  Akinesia=3  Dyskinesia =4 |
| Contractile function in segment 3 (mm) | Seg_3 | Normal= 1  Hypokinesia =2  Akinesia=3  Dyskinesia =4 |
| Contractile function in segment 4 (mm) | Seg_4 | Normal= 1  Hypokinesia =2  Akinesia=3  Dyskinesia =4 |
| Contractile function in segment 5 (mm) | Seg_5 | Normal= 1  Hypokinesia =2  Akinesia=3  Dyskinesia =4 |
| Contractile function in segment 6 (mm) | Seg_6 | Normal= 1  Hypokinesia =2  Akinesia=3  Dyskinesia =4 |
| Contractile function in segment 7 (mm) | Seg_7 | Normal= 1  Hypokinesia =2  Akinesia=3  Dyskinesia =4 |
| Contractile function in segment 8 (mm) | Seg_8 | Normal= 1  Hypokinesia =2  Akinesia=3  Dyskinesia =4 |
| Contractile function in segment 9 (mm) | Seg_9 | Normal= 1  Hypokinesia =2  Akinesia=3  Dyskinesia =4 |
| Contractile function in segment 10 (mm) | Seg_10 | Normal= 1  Hypokinesia =2  Akinesia=3  Dyskinesia =4 |
| Contractile function in segment 11 (mm) | Seg_11 | Normal= 1  Hypokinesia =2  Akinesia=3  Dyskinesia =4 |
| Contractile function in segment 12 (mm) | Seg_12 | Normal= 1  Hypokinesia =2  Akinesia=3  Dyskinesia =4 |
| Contractile function in segment 13 (mm) | Seg_13 | Normal= 1  Hypokinesia =2  Akinesia=3  Dyskinesia =4 |
| Contractile function in segment 14 (mm) | Seg_14 | Normal= 1  Hypokinesia =2  Akinesia=3  Dyskinesia =4 |
| Contractile function in segment 15 (mm) | Seg_15 | Normal= 1  Hypokinesia =2  Akinesia=3  Dyskinesia =4 |
| Contractile function in segment 16 (mm) | Seg_16 | Normal= 1  Hypokinesia =2  Akinesia=3  Dyskinesia =4 |
| Contractile function in segment 17 (mm) | Seg_17 | Normal= 1  Hypokinesia =2  Akinesia=3  Dyskinesia =4 |
| Right ventricular end-systolic area (cm^2^) | RV_ESA |  |
| Right ventricular end-diastolic area (cm^2^) | RV_EDA |  |
| Global longitudinal strain in the RV free wall (%) | GLS_RV |  |
| Global longitudinal strain segment 1 (%) | GLS_1 |  |
| Global longitudinal strain segment 2 (%) | GLS_2 |  |
| Global longitudinal strain segment 3 (%) | GLS_3 |  |
| Global longitudinal strain segment 4 (%) | GLS_4 |  |
| Global longitudinal strain segment 5 (%) | GLS_5 |  |
| Global longitudinal strain segment 6 (%) | GLS_6 |  |
| Global longitudinal strain segment 7 (%) | GLS_7 |  |
| Global longitudinal strain segment 8 (%) | GLS_8 |  |
| Global longitudinal strain segment 9 (%) | GLS_9 |  |
| Global longitudinal strain segment 10 (%) | GLS_10 |  |
| Global longitudinal strain segment 11 (%) | GLS_11 |  |
| Global longitudinal strain segment 12 (%) | GLS_12 |  |
| Global longitudinal strain segment 13 (%) | GLS_13 |  |
| Global longitudinal strain segment 14 (%) | GLS_14 |  |
| Global longitudinal strain segment 15 (%) | GLS_15 |  |
| Global longitudinal strain segment 16 (%) | GLS_16 |  |
| Global longitudinal strain segment 17 (%) | GLS_17 |  |
| Time date comments | Time_date_comments |  |
| Other comments | Other_comments |  |
